# Markers Predicting Cure With Combinatorial Treatment in a Mouse Model of Latent Autoimmune Diabetes in Adults

**DOI:** 10.1002/mco2.70813

**Published:** 2026-07-12

**Authors:** Wisal Sawaed, Ahmad Dallasheh, Sivan Eliyahu, Nura Aburomi, Aviad Sivan, Marina Kurtz, Michael Assa, Assaf Malka, Shira Perez, Ron Piran

**Affiliations:** ^1^ The Regenerative Medicine and Diabetes Laboratory The Azrieli Faculty of Medicine Bar‐Ilan University Safed Israel; ^2^ The Genomic Center The Azrieli Faculty of Medicine Bar‐Ilan University Safed Israel; ^3^ The Microscopy Center The Azrieli Faculty of Medicine Bar‐Ilan University Safed Israel

**Keywords:** β‐cell regeneration, autoimmunity, autoimmune diabetes, diabetes cure, T regulatory cells

## Abstract

Diabetes encompasses a range of diseases characterized by chronic hyperglycemia and serious health complications. However, predicting therapeutic response in latent autoimmune diabetes in adults (LADA) remains a major challenge, limiting the development of personalized treatment strategies. LADA is a relatively newly defined diabetes type that shares features of Type 1 diabetes (T1D) and Type 2 diabetes and is estimated to be more prevalent than T1D. We developed a LADA model in NOD mice and assessed combination treatment (CT) comprising GABA, sitagliptin, and omeprazole. Surprisingly, ∼30% of the CT‐treated mice completely recovered, exhibiting normoglycemia and insulin independence. We identified two cell‐free RNA markers, Adgrb1 and Chd5, that distinguish responders from nonresponders, indicating promising predictive ability. These discoveries offer a potential diagnostic tool for identifying LADA patients who could benefit from CT, representing an advance in personalized diabetes treatment. CT‐induced β‐cell neogenesis involved replication, providing valuable insights into β‐cell regeneration mechanisms. Furthermore, the cured mice exhibited insulitis primarily populated by T regulatory Type 1 cells, potentially suppressing autoimmunity and facilitating β‐cell survival and regeneration. This study opens new avenues for targeted LADA therapies and paves the way for precision medicine in diabetes management.

## Graphical abstract

This study explores a combinatorial treatment (CT) of GABA, sitagliptin, and omeprazole in a latent autoimmune diabetes in adults (LADA) mouse model. ∼30% of CT‐treated mice fully recovered, showing normoglycemia and insulin independence. Cell‐free RNA biomarkers Adgrb1 and Chd5 identify CT responders, aiding personalized treatment. Tr1 regulatory cells and CT‐induced β‐cell neogenesis suggest potential for targeted LADA therapies.

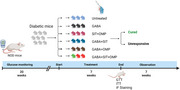

## 1 Introduction

Diabetes mellitus includes diseases characterized by chronic hyperglycemia due to either autoimmune β‐cell destruction, as in Type 1 diabetes (T1D) [1] or impaired β‐cell function, as in Type 2 diabetes (T2D) [2]. All diabetes types can lead to serious complications, including kidney disease, cardiovascular disease, blindness, and neuropathy [3, 4]. Latent autoimmune diabetes in adults (LADA) is a recently classified diabetes subtype that shares autoimmune characteristics with T1D but typically presents later in life, usually in individuals aged 30 years or older, often resembling T2D [5]. Unlike T1D's rapid progression [6], LADA advances slowly; if progression is rapid, a T1D diagnosis is usually made [7]. LADA may also exhibit T2D‐associated traits, like weight gain and hypertension [8], and generally requires insulin therapy early in the treatment regimen [9]. However, LADA is frequently misdiagnosed as T2D, leading to suboptimal treatment [5]. It is estimated that a substantial portion of lean T2D patients are, in fact, LADA patients [10]. Accordingly, it is hypothesized that LADA patients establish ∼10% of all T2D patients. Considering that T2D patients constitute approximately 90% of all diabetic patients and that T1D patients make up approximately 8%, LADA is more prevalent than T1D is (10 × 90/100 = 9% of total diabetic patients) [11].

Over time, all types of diabetes ultimately lead to the loss of pancreatic β‐cells, resulting in decreased insulin secretion, leading to excessive glucose production and decreased cellular uptake of glucose [12]. Consequently, developing novel strategies to protect, regenerate, and restore β‐cells represents a promising therapeutic avenue for all diabetes patients. Several drugs, including γ‐aminobutyric acid (GABA), dipeptidyl peptidase IV inhibitors (DPP‐4i), such as sitagliptin (SIT), and proton pump inhibitors (PPIs), such as omeprazole (OMP), have been found to potentially stimulate β‐cell proliferation and enhance β‐cell function.

GABA, a major neurotransmitter in the central nervous system (CNS), serves as a paracrine signaling molecule, metabolic intermediate, and trophic factor [13]. GABA is produced not only in the CNS but also at several extraneural sites, including pancreatic β‐cells and immune cells [14]. In the pancreas, GABA plays multiple roles. It is believed to participate in the regulation of pancreatic hormone secretion by acting as a fast paracrine signaling molecule, facilitating communication between β‐cells and other islet cells [15]. Additionally, GABA has been shown to promote β‐cell growth and survival, suppress inflammation, and increase the number of regulatory T cells (T‐regs). In mouse models of T1D, GABA treatment reduced islet lymphocytic infiltration, restored β‐cell mass, reversed hyperglycemia, and resulted in increased serum insulin, decreased glucagon concentrations, and improved glucose homeostasis [12]. Furthermore, studies by Ben‐Othman et al. reported that GABA induces α‐cell‐mediated β‐like cell neogenesis in vivo [16]. SIT, a DPP‐4 inhibitor, primarily treats T2D by preventing degradation of incretin hormones (GLP‐1 and GIP), which stimulate insulin secretion and β‐cell proliferation [17]. DPP‐4 inhibition has demonstrated β‐cell protective effects and improved glucose control in T1D and T2D. In the immune system, it increased T‐regs and CD4+ T‐cell migration, enhancing islet graft survival in NOD mice [18, 19, 20, 21]. OMP, a common PPI, treats conditions related to excess stomach acid. PPIs indirectly increase serum gastrin levels through negative feedback [22]. The gastrin hormone is released by G‐cells found in the stomach, duodenum, and pancreas. Gastrin stimulates β‐cell neogenesis in the pancreatic ductal complex, β‐cell replication, and improves glucose tolerance in animal models [23]. Recent discoveries have revealed the beneficial effects of PPIs on glycemic control, particularly in T2D patients, which are likely attributed to increased gastrin levels [24, 25]. Furthermore, treatment with OMP leads to elevated insulin levels and improved hyperglycemic control in T2D patients [17, 26].

Combining GABA and SIT has shown promise in improving blood glucose and insulin levels, promoting β‐cell proliferation, regeneration, and reducing apoptosis [27, 28]. Similarly, DPP‐4i and PPI cotherapy increased GLP‐1 and gastrin levels, restoring pancreatic β‐cell mass in NOD mice [29, 30, 31]. Recent studies suggest a combination of GABA, SIT, and OMP may benefit T1D [32, 33]. In this study, we present a newly developed LADA model in NOD mice, demonstrating that combinatorial treatment (CT) with GABA, SIT, and OMP significantly improved diabetic symptoms. Remarkably, approximately 30% of CT‐treated mice were completely cured of diabetes, maintaining normoglycemia for 7 weeks after the last drug administration, marking an unprecedented achievement in managing this otherwise irreversible disease. Moreover, we successfully identified cell‐free RNA (cfRNA) molecules that characterize the remaining 70% of mice that did not respond to the treatment (unresponsive individuals), that is, those that remained hyperglycemic despite CT, pointing to a potential diagnostic tool for identifying successful responders to CT.

## 2 Results

### 2.1 CT is Relevant to an NOD Mouse Model of LADA‐Like, Slowly Progressing Autoimmune Diabetes

Autoimmune diabetes in NOD mice is usually very rapid and violent, making them an ideal T1D animal model [34, 35]. The majority of NOD mice develop T1D at an early age [36, 37]. When we administered CT to diabetic NOD mice, most exhibited a minimal response (Figure S1A). However, in this preliminary experiment, there was one exceptional case in which a NOD mouse exhibited T1D onset at the age of 31 weeks. This mouse received CT for 45 days and demonstrated a remarkable and complete recovery from diabetes. This unique finding led us to focus our efforts on addressing LADA, and subsequent follow‐up studies in additional mice were conducted using aged mice exceeding 20 weeks of age.

### 2.2 CT Induces Remission in an NOD Mouse Model of LADA

**FIGURE 1:**
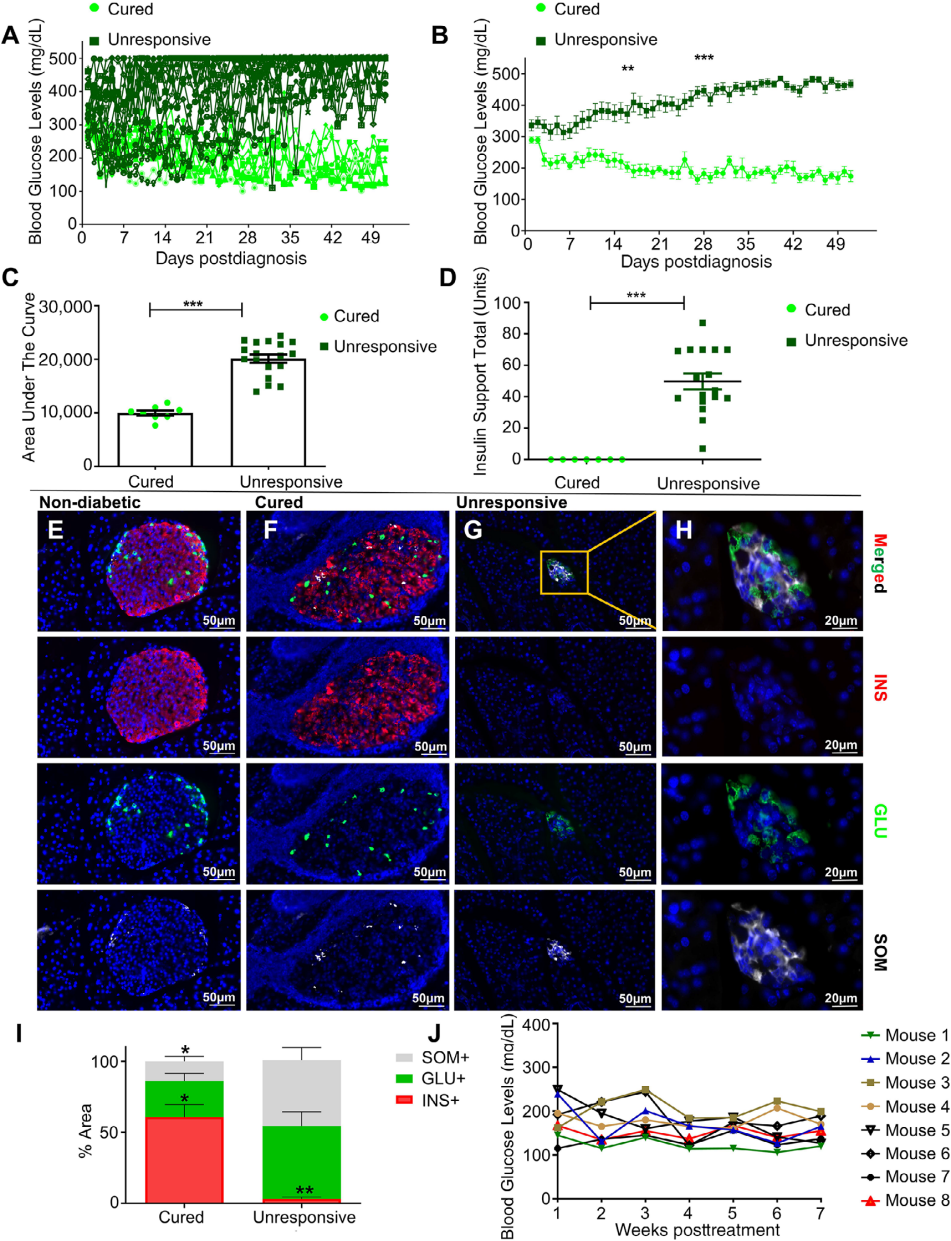
Oral treatment with CT induces long‐term normoglycemia in 30% of NOD mice. (A) Real‐time glycemic levels of individual mice (*n* = 26, 8 = number of cured mice—light green, 18 = number of unresponsive mice—dark green) treated with CT. Each line represents a CT‐treated mouse. Starting on Day 30, a bimodal distribution emerged, indicating cured and unresponsive mice to CT. (B) The average glycemic levels of the two groups presented in panel (A). (C) The area under the curve (AUC) of the graphs presented in panel (A). (D) Insulin was administered to support normoglycemia in diabetic mice. Total insulin amounts (units) are indicated for each mouse. Please note that cured mice did not require any insulin support. (E–H) Representative immunofluorescence images of pancreatic islets showing insulin staining (red), glucagon staining (green), and somatostatin staining (white) in the three mouse groups. (E) A control islet from a nondiabetic NOD mouse. Note the relative ratio of β‐cells within the islet. (F) An islet from a cured mouse. Note that the relative ratio of β‐cell complements the normal ratio. (G and H) A low magnification (G) and a high magnification (H) of an islet from an unresponsive mouse. Note that β‐cells are practically absent in these islets. (I) %Area quantification of the different endocrine cells composing the islets in cured versus unresponsive mice. Note that in cured mice β‐cell mass increased. (J) The cured animals were followed for additional 7 weeks posttreatment with no medication at all (no insulin support and no other drugs). Each line represents one mouse (*n* = 8). Note that all animals remained normoglycemic. Scale bars in E–G = 50 µm and H = 20 µm. The data are presented as the mean ± standard error of the mean (SEM). The statistical significance of the differences was determined using repeated‐measures ANOVA. *Indicates *p < 0.05, **p < 0.01, ***p < 0.001.

To further investigate the effectiveness of CT for treating LADA, we selected nondiabetic NOD mice older than 20 weeks and monitored them twice weekly for the onset of diabetes. CT was administered to mice that exhibited two consecutive hyperglycemic measurements for 7 weeks (Figure S1B). Of the 26 mice receiving CT, eight exhibited a significant reduction in glycemic levels and achieved complete insulin independence, representing a 30% recovery rate (Figure 1A–D). Therefore, we first aimed to characterize the islets of the cured mice, and later aimed to study what differentiates the cured (30%) from those that remained hyperglycemic, which we termed unresponsive (70%).

Unresponsive mice were sacrificed immediately after the 7‐week CT period, while the cured animals were monitored for an additional 7 weeks without treatment to confirm their complete recovery and to monitor for long‐term effects. It was calculated that a 7 week monitoring could be equivalent to 4.67–5.4 human years [38, 39, 40]. When addressing the specific age of the mice in our experiment (20 weeks of age and older), the 7 week monitoring period is equivalent to almost 19 years [38, 39]. All the cured mice maintained blood glucose levels below 250 mg/dL, indicating sustained insulin independence (Figure 1D,J). We isolated and examined the pancreata to assess the endocrine cell content (Figure 1E–I).

**FIGURE 2:**
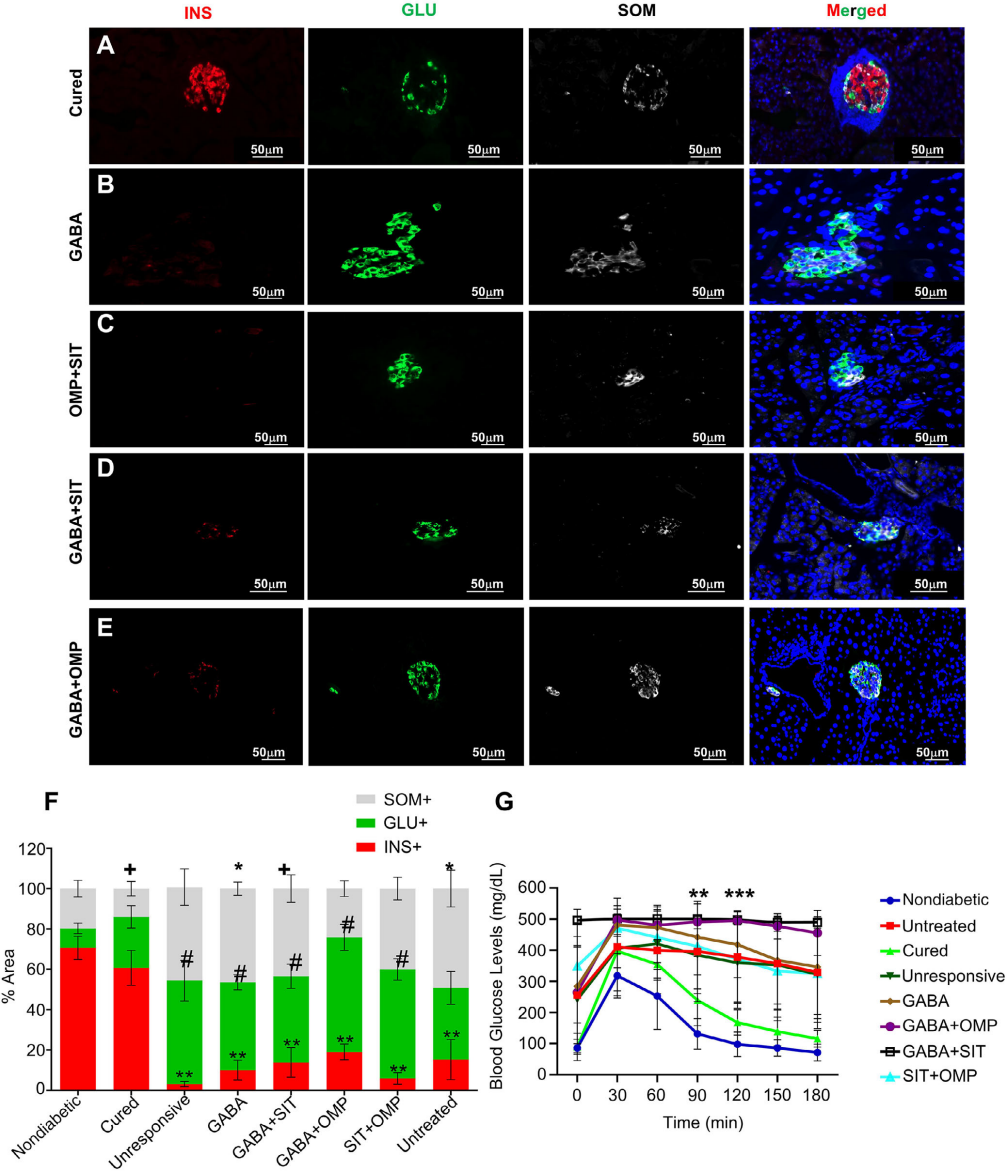
Only nondiabetic and cured mice presented high β‐cell masses. Islets from a cured mouse (A), a GABA‐treated mouse (B), an OMP+SIT (C), a GABA+SIT (D), and a GABA+OMP (E). Note that only cured mice regained β‐cell mass as indicated by INS+ cells. Insulin staining is shown in red, glucagon staining in green, and somatostatin staining in white. (F) Quantitative analysis of % endocrine cell types in islets from the different experimental groups (cured and unresponsive values are identical to the graph presented in Figure 1I). Only cured mice presented complementary INS+ levels to nondiabetics. (G) An intraperitoneal glucose tolerance test (IPGTT) was performed on the mice after they reached the end of the 7‐week treatment. Only cured mice showed glucose responsive behavior, similarly to nondiabetic mice, as was also indicated by β‐cell mass quantification in panel (F). The scale bars in panels (A–E) = 50 µm. The error bars are SEM. The statistical significance of the differences was determined using repeated‐measures ANOVA. *Indicates *p < 0.05, **p < 0.01, ***p < 0.001. *—nondiabetic and cured in comparison with all the other groups. #—in comparison with the nondiabetic group. +—in comparison with the cured group.

In the cured mice that received CT, the percentage of β‐cells reached 60% of the total endocrine cells, which was comparable to the percentage observed in nondiabetic mice (Figure 1F, quantified in I compared with 70% in nondiabetic mice; Figure 2F). In contrast, the other groups presented lower amounts of insulin‐positive β‐cells (3% unresponsive, 10% GABA, 13% GABA+SIT, 19% GABA+OMP, 6% SIT+OMP, 15% untreated; quantified in Figure 2F). This was further supported by their inability to reduce glucose levels in glucose tolerance tests (GTTs; Figure 2G).

**FIGURE 3:**
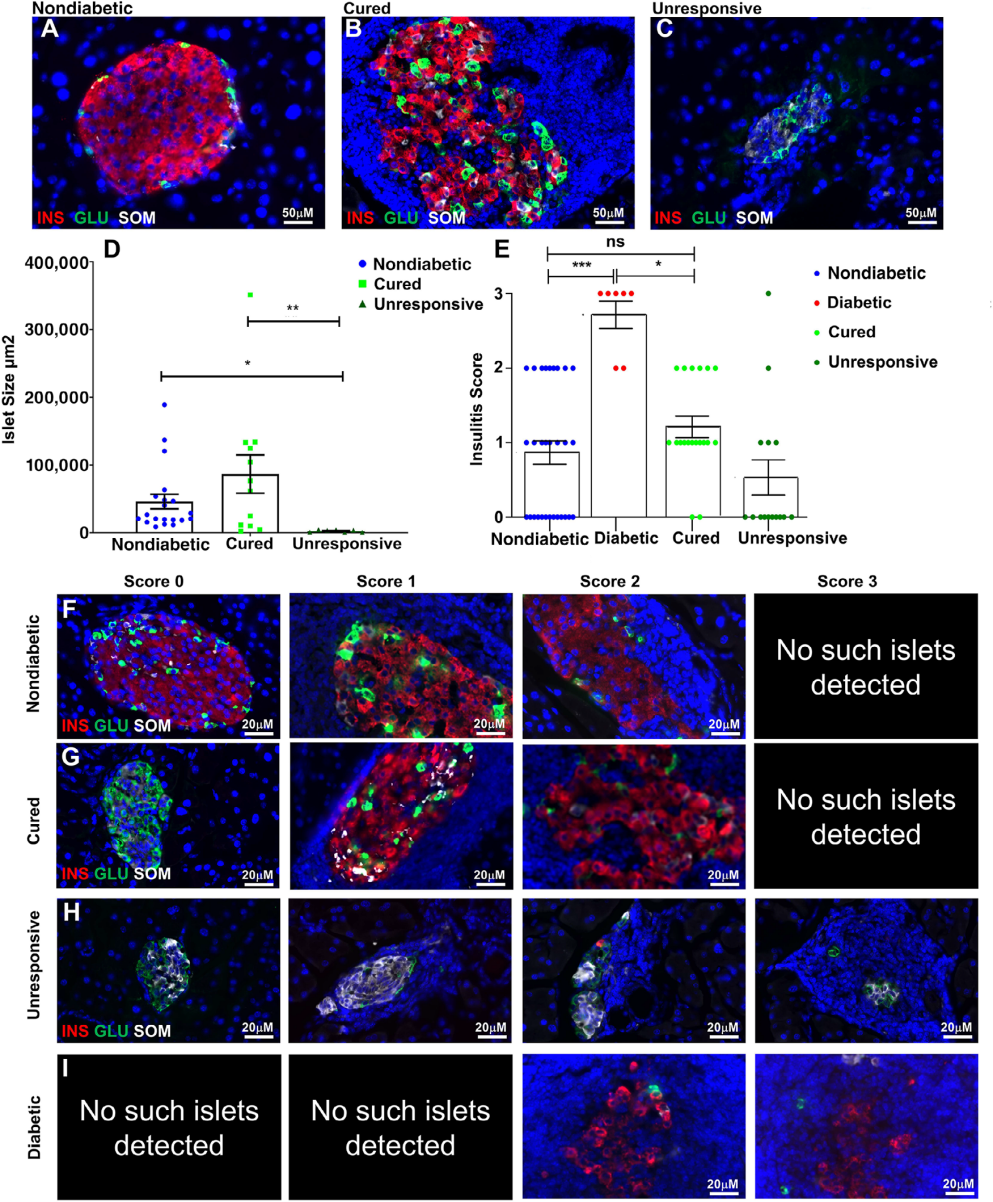
Cured mice had enlarged and atypical islets. Immunofluorescence images of pancreatic islets showing insulin staining in red, glucagon staining in green, and somatostatin staining in white in the three indicated mouse groups. (A) An islet from a nondiabetic NOD mouse. (B) An islet from a cured mouse. A full‐size image of panel (B) is in Figure S2A. Note that this islet is considerably larger than the one in panel (A). (C) An islet from an unresponsive mouse. This islet is smaller and lacks insulin expression. (D) Quantification of islets size. The islets in the cured group were larger than those in the other groups as determined using repeated‐measures ANOVA. (E) Scatter dot plot analysis of the histological scores of different islets taken from mice that received CT (cured and unresponsive) compared with those of nondiabetic and diabetic mice, according to the following criteria: 0: no infiltration. 1: Peri‐insulitis. 2: Infiltrative insulitis <50% of the islet. 3: Severe infiltrative insulitis >50% of the islet. Statistical significance was determined by Kruskal–Wallis test. Immunofluorescence images of pancreatic islets showing the difference in insulitis morphology between the groups. (F) Islets from nondiabetic NOD mice. Note that islets corresponding to scores 0–2 but no score 3 were found. (G) Islets from cured mice. Full size images of INS+ islets are in Figure S2B and C, respectively. Note that islets corresponding to scores 0–2 but no score 3 were found. (H) Islets from unresponsive mice. Here, we were able to identify score 3 islets. Note that in all islets no INS+ β‐cells are found. (I) Islets from diabetic mice. Note that here only score 2 and 3 islets were observed. The allocated box remains empty stating “No such islets detected” If no islets are found in a specific category. The scale bars in panels (A–C) = 50 µm and in panels (F–I) = 20 µm. The data are presented as the mean ± standard error of the mean (SEM). The statistical significance of the differences is indicated by *p < 0.05, **p < 0.01, ***p < 0.001, ns—not significant.

As maintaining an appropriate balance of α‐, β‐, and δ‐cells may play an important role in preventing hypoglycemic episodes, we checked the composition of the islet cell types in each experimental group. Nondiabetic and cured mice exhibited lower percentages of α‐ and δ‐cells (for glucagon: 9.6 and 25%, respectively; for somatostatin: 19.6 and 13.9%, respectively; quantified in Figure 2F) compared with the control groups (for glucagon: 51% unresponsive, 43% GABA, 42% GABA+SIT, 56% GABA+OMP, 54% SIT+OMP, and 35% untreated; for somatostatin: 46% unresponsive, 46% GABA, 43% GABA+SIT, 24% GABA+OMP, 40% SIT+OMP, and 49% untreated). All the cured animals exhibited large islets (quantified in Figure 3D) surrounded by leukocyte infiltrates (insulitis; Figures 1F, 2A, and 3B,G, with wider view images in Figure S2A–C, respectively), while these infiltrations were largely absent in nondiabetic and unresponsive NOD mice (Figure 1E,G,H, as well as Figure 3A,C). It is possible that the insulitis had already resolved in unresponsive mice at this stage of the disease (7 weeks post onset), as previously demonstrated [41].

### 2.3 CT Yields Comparable Outcomes in NZO and NOD Mouse Models

Given that CT did not achieve complete recovery in all NOD mice, we explored the possibility of using NZO mice, which develop diabetes at a later age and might be a more suitable model for LADA. However, CT yielded similar recovery rates in NZO mice (three out of nine mice; a 33% cure rate; Figure S3). This comparative analysis indicates that the NZO experiments did not yield results different from those obtained in the NOD model, which we ultimately focused on.

### 2.4 Only CT Achieves Remission; Subsets of Drugs Are Less Effective

As previous studies reported that GABA, SIT, and OMP alone were sufficient for achieving insulin independence and normoglycemia [32, 42, 43], it was important to validate the necessity of all CT components. We tested GABA, GABA+SIT, GABA+OMP, and SIT+OMP treatments (Figure S4). None of these combinations were as effective at reducing blood glucose levels as was CT. Furthermore, only CT‐treated mice displayed a bimodal distribution of blood glucose levels (Figure S4A). Histological examination of the pancreata from the different treatment groups did not reveal the same insulitis morphology observed in the CT group. In the non‐CT groups, the islets, which consisted mainly of α‐ and δ‐cells, remained shrunk (Figure 2B–E). Additionally, only CT‐cured mice, like nondiabetic NOD mice, exhibited a recovery response in the GTT (Figure 2G).

### 2.5 Chd5 And Adgrb1 Levels Correlate With CT Outcome in LADA‐Modeled Mice

**FIGURE 4:**
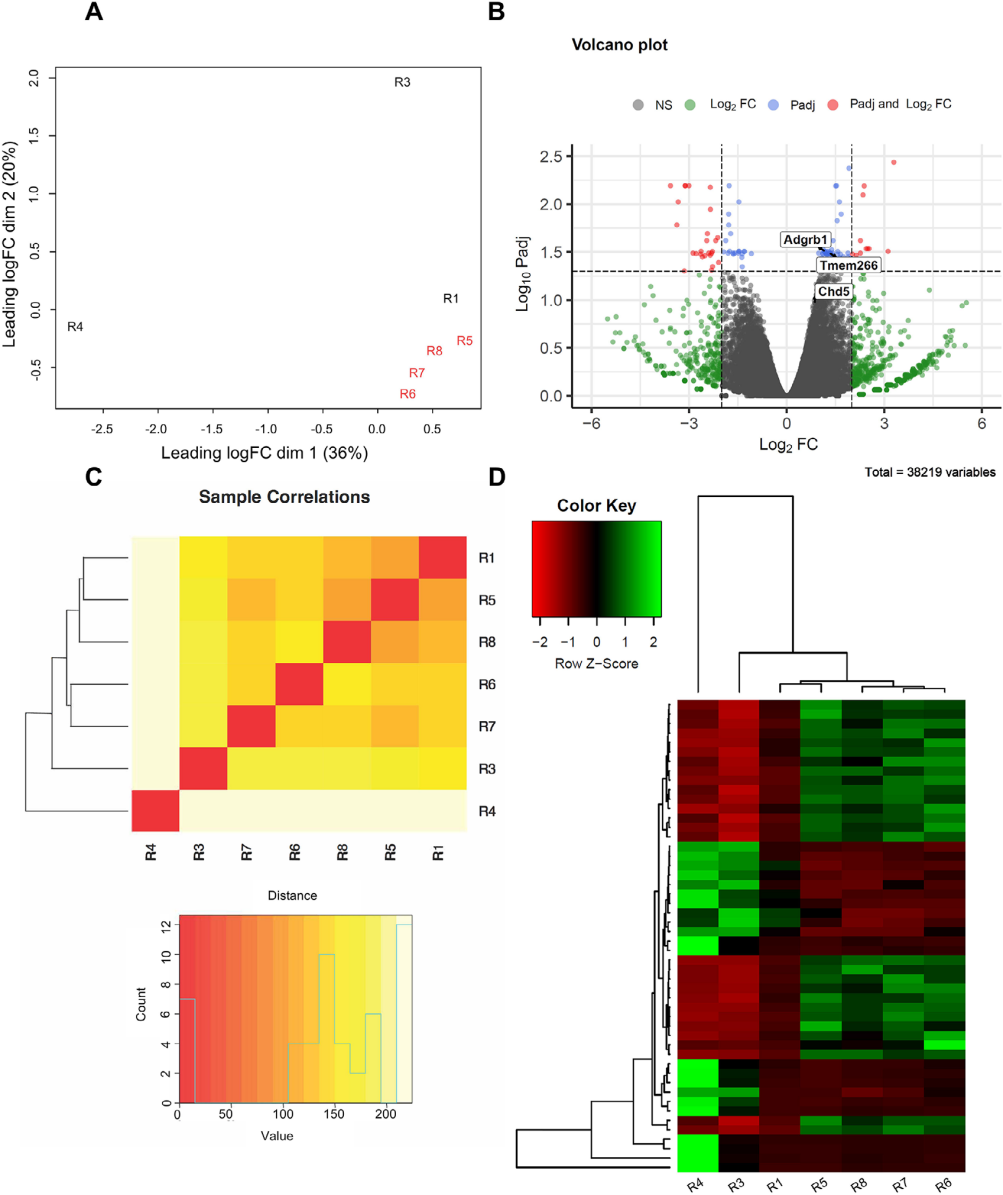
Cell‐free RNA differentiates cured and unresponsive mouse populations before treatment. (A) A two‐dimensional sample relation based on multidimensional scaling (MDS). The distance between two samples was interpreted as the log fold change between the two samples for the top 500 genes (red, unresponsive; black, cured). (B) Volcano plot comparing the results of the differential expression analysis between cured and unresponsive mice. log_2_FC, and adjusted or unadjusted *p* values. The cutoff for log_2_FC was >|1|. (C) A heatmap based on normalized counts per million (CPM) read counts. (D) A heatmap visualizing the top 50 DE genes between cured and unresponsive mice showing the expression patterns of the genes across samples, clustering together genes that have correlated expression patterns. The heatmap clusters samples based on the Euclidean distance between the expression values. The detailed version of (D) with gene names is in Figure S7.

To differentiate CT‐cured mice from unresponsive mice, we repeated the CT experiment this time by obtaining blood samples from all mice after diabetes onset, but before starting treatment (*n* = 13). After the treatment, as described earlier, four out of 13 mice recovered. We then returned to the blood samples and arranged them according to the cured/unresponsive status. Of these, four cured and four unresponsive (randomly chosen) mouse samples were taken for analysis. Circulating cfRNA was isolated as described in *Materials and Methods* section, after which libraries were generated. To verify that the library was from a mouse origin, a subset of each sample was mapped to different genomes, including the mouse genome (Fastq‐screen), resulting in most of the reads uniquely mapping to the mouse genome (Figures S5 and S6). The R2 sample appeared clustered together with the unresponsive group, contradicting the sample origin; thus, we decided to eliminate the “R2” sample from the MDS, heatmap, and dendrogram plots (Figure 4A,D; a detailed heatmap is in Figure S7.).

Furthermore, we extracted the differentially expressed genes that had a fold change greater than 2 and an adjusted *p* value lower than 0.05 (multiple testing was corrected using a false discovery rate of 5%), which resulted in 116 genes (in the volcano plot; Figure 4B).

**FIGURE 5:**
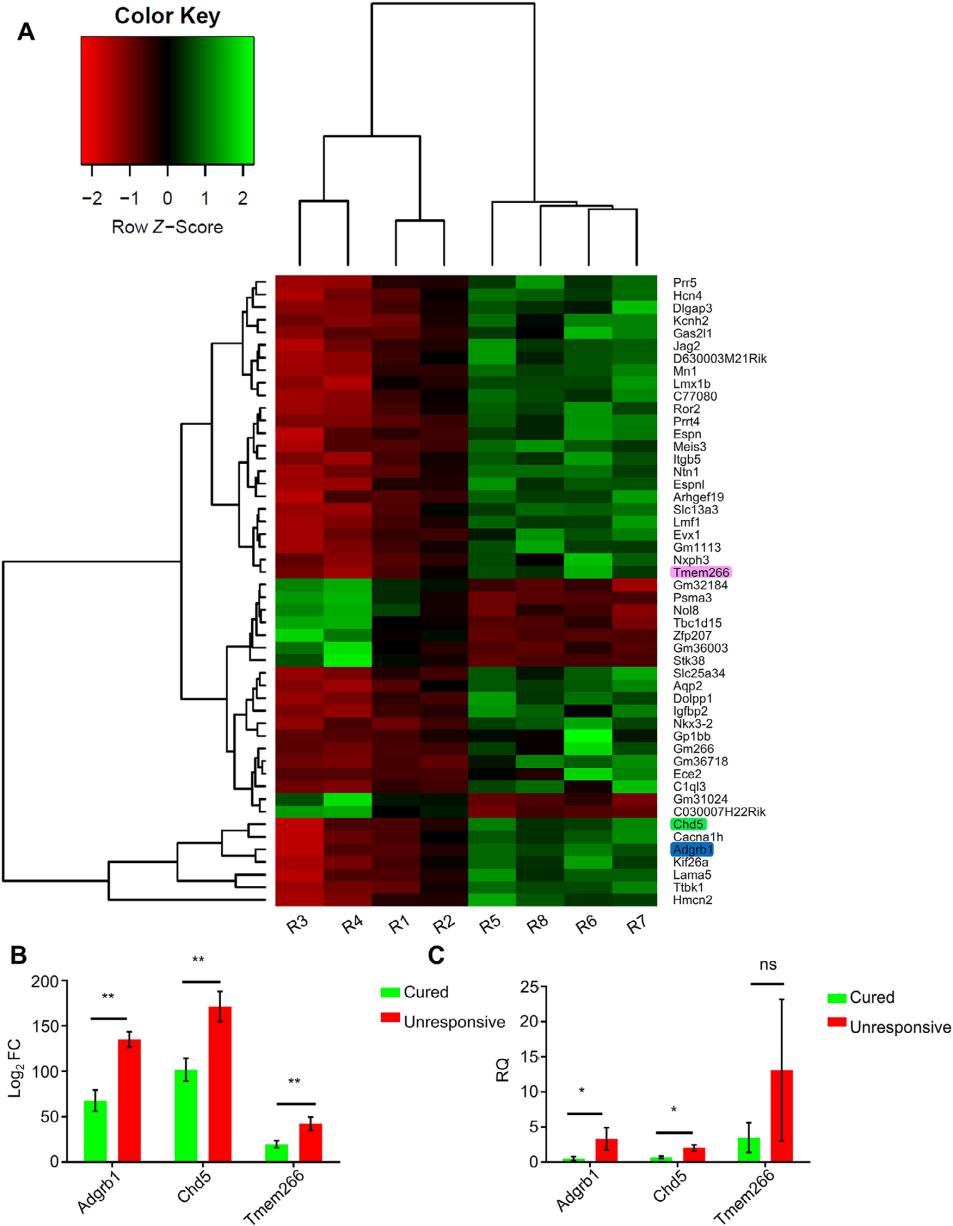
The levels of the cell‐free RNA markers Adgrb1 and CHD5 were used to differentiate between cured and unresponsive mice and predict the success of CT. (A) Heatmap analysis showing genes differentially expressed between cured (R1–R4, R2 is now included) and unresponsive (R5–R8) mice. Green or red indicates differentially upregulated or downregulated genes, respectively. The Adgrb1, Chd5, and Tmem266 genes exhibited significant differential expression and were further analyzed using RNA sequencing (RNA‐seq) and quantitative real‐time PCR (qRT‐PCR). (B) RNA‐seq results for the selected genes Adgrb1, Chd5, and Tmem266 showing that their levels were greater in unresponsive animals than in cured animals. (C) qRT‐PCR analyses of the Adgrb1, Chd5, and Tmem266 genes. For Chd5 and Adgrb1, the results were consistent with the sequencing data; however, for Tmem266, the deviation was large. Error bars represent SEM. *T* tests were used to determine the statistical significance of the differences. *Indicates *p* < 0.05, ** indicates *p* < 0.01, and ns indicates not significant.

However, one must note that R2 is not an experimental outlier. Animal R2 responded successfully to the treatment and remained normoglycemic for 7 additional weeks. Therefore, we returned to the heatmap, this time including R2 back in the analysis, and we searched for gene markers that displayed R2 genes together with R1, R3, and R4 and separated it from R5‐8 (Figure 5A). When R2 was excluded from the heatmap, its dendrogram clustered all unresponsive animals in a group, together with R1, while the cured animals were not clustered at all (Figure 4D). However, when we reincorporated R2 into the heatmap, the dendrogram clustered the unresponsive mice into one group and the cured mice into the other (Figure 5A).

To enhance the translational relevance of our findings and facilitate the development of a potential diagnostic tool, we sought to identify circulating blood markers capable of distinguishing potential responders to LADA CT. We examined both biochemical blood indicators (data not shown) and cfRNA extracted from plasma. Circulating cfRNA was isolated from peripheral blood samples obtained as liquid biopsies. We established two guidelines for gene marker selection: (a) that the differences between the two groups should be uniform for all samples and (b) that the deviation in gene expression within the group should be minimal. Genes that were mapped to specific and relevant tissues and were significantly related to that tissue were eventually selected. From this newly generated heatmap analysis, we selected three genes, Chd5, Adgrb1, and Tmem266, for further analysis (Figure 5A,B).

We then verified the circulating RNA expression levels of these genes using quantitative reverse transcription PCR (qRT‐PCR). The results for Chd5 and Adgrb1 were consistent with the sequencing data; however, for Tmem266, the deviation was large (Figure 5C).

### 2.6 Cured Mice Exhibit Nondestructive Insulitis Patterns

Cured NOD mice presented islets with insulitis (Figures 1F, 2A, and 3B). However, it is reported that Insulitis in NOD mice leads to β‐cell loss in the islets of Langerhans as the disease progresses [41]. Therefore, we revisited Figure 3. Histological scoring was performed as described previously [44]. The islet and insulitis scores of the sections were reviewed according to the following criteria: 0: no infiltration. 1: Peri‐insulitis—in which immune cells surround the islet of Langerhans but do not infiltrate it. 2: Infiltrative insulitis penetrates <50% of the islet by leukocytes, yet no individual β‐cells are engulfed completely by immune cells. 3: Severe infiltrative insulitis occurs when >50% of the islet is completely engulfed by immune cells, including many encircled individual β‐cells. In cured NOD mice, histological scoring revealed that most of the islets had scores of 1 or 2, that is, less than 50% insulitis. In addition, there were a few islets without insulitis and we did not identify islets with severe insulitis. Moreover, there were more islets with a score of 1 than in the other groups. However, in unresponsive mice, there were many islets without insulitis (score 0), as we did not find any surviving β‐cells in those islets at this progressive stage of the disease. In normal diabetic animals (1 week after disease onset), most of the islets exhibited severe insulitis (more than 50% infiltration within the islet), as expected (Figure 3E–I).

### 2.7 Cured Mice Exhibit Notably Large Islets

We noticed a unique observation regarding the size of the islets, similarly to the report by Ben‐Othman et al. [16], cured mice had larger islets than did normal mice (Figure S2A—compared with Figure 3A,F; note the scale bar size). In unresponsive mice, islets were depleted of β‐cells, and thus, their size was dramatically decreased (Figure 3C,H, quantified in D).

### 2.8 Insulitis in Cured Mice is Enriched With T Regulatory Type 1 Cells That May Support β‐Cell Regeneration

**FIGURE 6:**
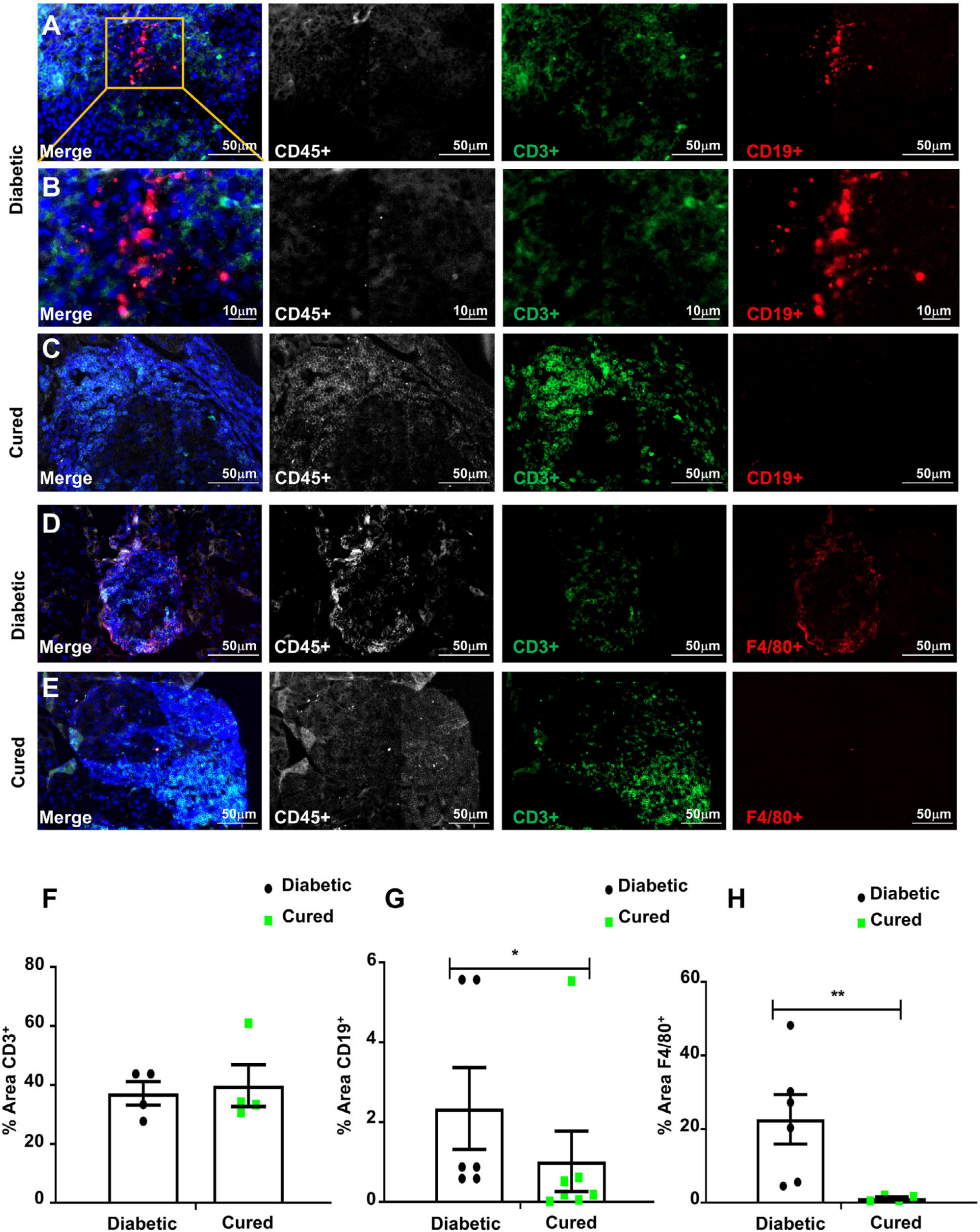
Cured mouse insulitis comprises almost exclusively T cells. Immunofluorescence images of pancreatic islets showing CD45+ staining (white), CD3+ staining (green), and CD19+ staining (red) (A–C) or F4/80+ staining (red) (D and E). (A and B) A low magnification (A) and a high magnification (B) image of an islet from a diabetic mouse. (C) An islet from a cured mouse. (D) An islet from a diabetic mouse. (E) An islet from a cured mouse. (F) Quantification of CD3+ cells. (G) Quantification of CD19+ cells. (H) Quantification of F4/80+ cells. Note that the insulitis in diabetic mice is composed of a mixture of T cells (indicated by CD3+ staining), B cells (indicated by CD19+ staining), and macrophages (indicated by F4/80+ staining). In comparison, the insulitis observed in cured mice contained almost exclusively T cells. Scale bars in panel (A), (C–E) = 50 µm; scale bars in panel (B) = 10 µm. The data are presented as the mean ± standard error of the mean (SEM). *T* tests were used to determine the statistical significance of the differences. *Indicates *p < 0.05 and ** indicates *p* < 0.01.

Cured mice exhibited normoglycemia and an almost normal GTT response (Figures 1A,B and 2G). However, we were surprised to see that their islets exhibited insulitis (Figures 1F and 3B,G with wider view images in Figure S2; quantified in Figure 3E), which persisted even 7 weeks after the 7‐week treatment. To understand this phenomenon, we compared the insulitis composition between cured mice and diabetic NOD mice. A diabetic NOD mouse's insulitis contained a mixture of T cells (indicated by CD3+ staining, 37.17%; Figure 6A,B,D; quantified in F), B cells (indicated by CD19+ staining, 2.34%; Figure 6A,B; quantified in G), and macrophages (indicated by F4/80+ staining, 22.66%; Figure 6D; quantified in H). However, B‐cell lymphocytes and macrophages were rare in the insulitis specimens of the cured mice (1.01 and 1.23%, Figure 6C,E; quantified in G and H, respectively).

**FIGURE 7:**
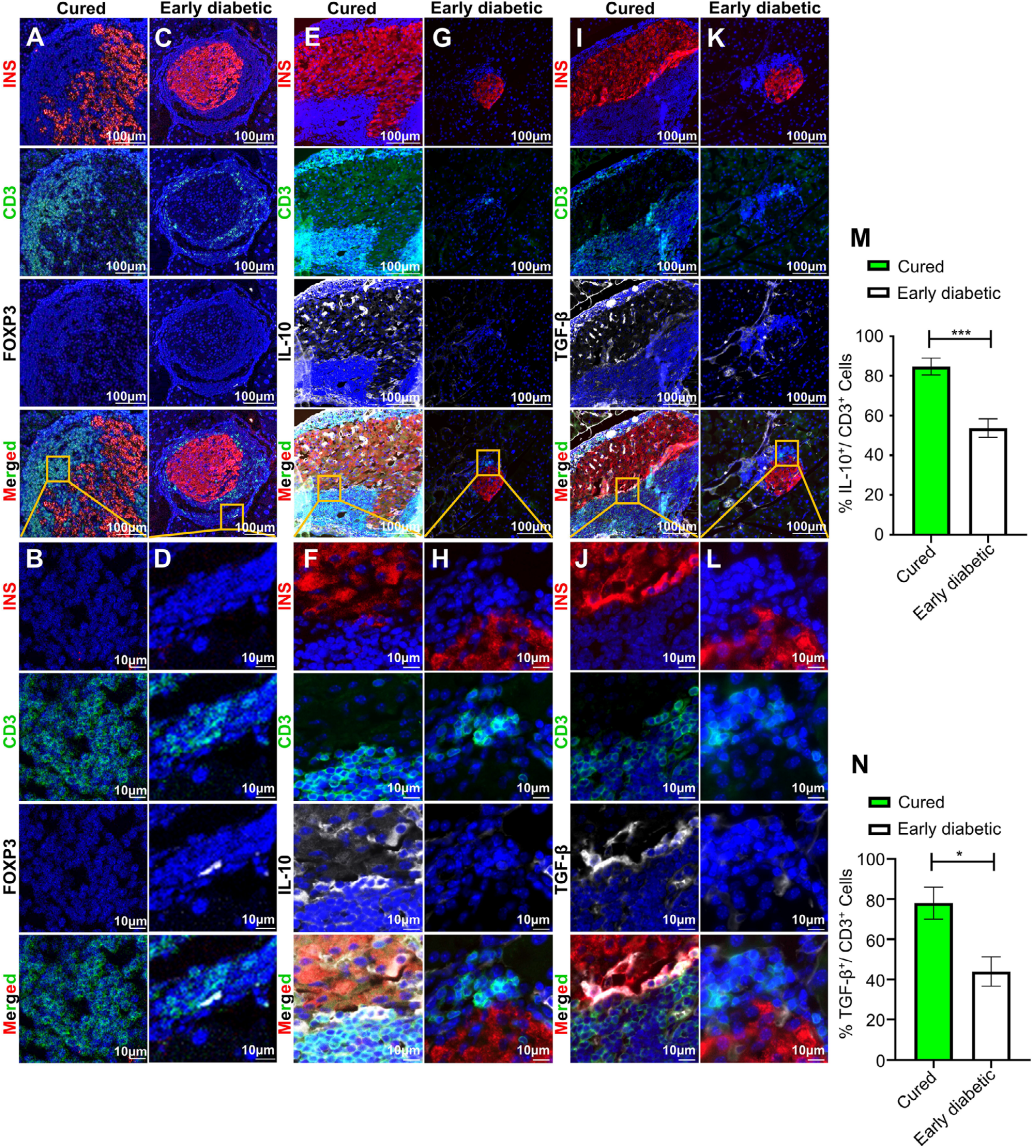
Tr1 cells, a FOXP3‐negative subpopulation of T‐regs, are the regulatory cells that suppress the immune response in the insulitis of cured mice. Representative immunofluorescence images of pancreatic islets showing insulin staining (red) and CD3 (a T‐cell marker) staining (green). (A–D) FOXP3, a classical T‐reg marker, is stained in white. (A and B) Low‐magnification (A) and high‐magnification (B) images of an islet from a cured mouse. No FOXP3+ cells were found in the cured islets. The full‐size image of this islet is presented in Figure S8A. (C and D) Low‐magnification (C) and high‐magnification (D) images of an islet with early insulitis obtained from an early diabetic mouse. (E–H) IL‐10 staining is shown in white. (E and F) A low magnification (E) and high magnification (F) image of an islet of a cured mouse. Note that most CD3+ T cells are also IL‐10+ cells. (G and H) Low‐magnification (G) and high‐magnification (H) images of an islet with early insulitis obtained from an early diabetic mouse. Note that most of the CD3+ T cells are also IL‐10^−^ cells. (I–L) TGF‐β staining is shown in white. (I and J) Low magnification (I) and high magnification (J) images of an islet from a cured mouse. Note that most of the CD3+ T cells are also TGF‐β+ cells. (K and L) Low‐magnification (K) and high‐magnification (L) images of an islet with early insulitis obtained from an early diabetic mouse. Note that most of the CD3+ T cells are also TGF‐β^−^ cells. Additional experimental groups are presented in Figure S8. (M) Quantification of the IL‐10+ cells/CD3+ cells ratio. In cured mice, this population reached >80% of the total T cells. (N) Quantification of TGF‐β+ cells/CD3+ cells. In cured mice, this population reaches ∼80% of the total T cells. The scale bars in panels (A, C, E, G, I, and K) = 100 µm, (B, D, F, H, J, and L) = 10 µm. *T* tests were used to determine the statistical significance of the differences. *Indicates *p < 0.05 and ***p < 0.001.

As cured mouse insulitis contains almost exclusively T cells and because these insulitis cases are likely inactive immunologically, we speculated that a large proportion of these T cells will be T‐regs. To identify T‐reg population, we stained for FOXP3, a classical T‐reg marker (Figures 7A,B and S8A–F). However, while the FOXP3 antibody is functional and as some FOXP3+ T cells were found in early insulitis—during the early diabetic stage and in unresponsive islets (Figure 7C, high magnification in D, and Figure S8E,F, respectively)—no FOXP3+ T cells were found in the large islets of the cured mice (Figure 7A, high magnification in B with wider view in Figure S8A,B). FOXP3 is considered the canonical T‐reg marker. However, not all T‐regs express the FOXP3 protein on their membranes [45, 46, 47]. One of these populations is the T regulatory Type 1 (Tr1) cells, a distinct FOXP3^−^ regulatory lineage characterized by high secretion of IL‐10 and TGF‐β and typically expressing surface molecules such as LAG‐3 and CD49b. Their in situ identification relies primarily on their cytokine profile. In this context, we detected IL‐10+ and TGF‐β+ CD3+ lymphocytes consistent with Tr1 cells within insulitis. Tr1 cells were found to populate ∼80% of the T cells in the cured mice (Figure 7E,I; high magnification in F and J; quantified in M and N). The fact that most T‐cells in cured mice are regulatory is an important finding. In the early diabetic stage, these percentages may reach only ∼40% (Figure 7G,K, quantified in M and N). The fact that β‐cells survive and proliferate in the presence of Tr1 cells indicates that Tr1 cells are good study targets for treating autoimmune diabetes.

### 2.9 Replication Drives β‐Cell Neogenesis in Cured Mice

**FIGURE 8:**
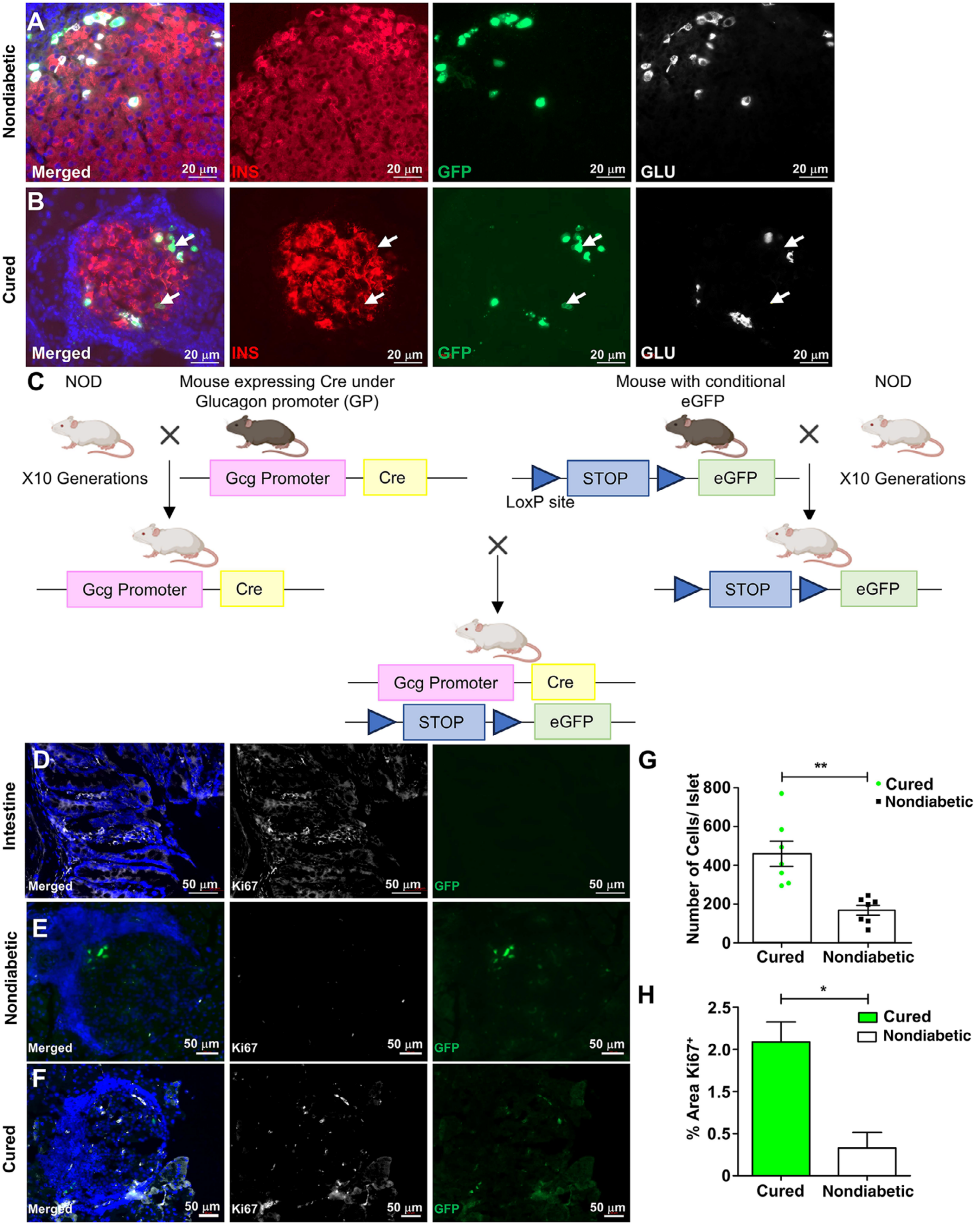
CT leads to β‐cell replication in cured mice. (A) An islet from a nondiabetic mouse. (B) An islet from a cured mouse. A few β‐cells appear in green in the cured mouse, an indication for an α‐ to β‐cell transdifferentiation. This indicates that β‐cell neogenesis was largely not from α‐cell progenitors. The arrows indicate cells double‐positive for GFP and insulin. For panels (A) and (B), insulin staining is shown in red, eGFP staining in green, and glucagon staining in white. (C) Schematic illustration of the creation of the glucagon–Cre driving the eGFP NOD mouse strain. (D) Intestines taken from a non‐diabetic mouse showing many Ki67+ cells. (E) An islet from a nondiabetic mouse. A few Ki67+ cells were observed. (F) An islet from a cured mouse. In the core of cured mouse islets, there are many Ki67+ cells, most of which are β‐cells. For panels (D–F), Ki67 staining is shown in white, and eGFP staining is shown in green. (G and H) quantification of the data presented in panels (E and F). (G) Greater INS+ area is a result of islet‐cells’ proliferation as indicated by the number of nuclei per islet and not the result of hypertrophy. (H) Cured mice represent higher Ki67+ cells per islet, indicating higher cell division in CT mice. Scale bars in panels (A and B) = 20 µm, panels (D and F) = 50 µm. *T* tests were used to determine the statistical significance of the differences. *Indicates *p < 0.05 and **p < 0.01.

In the unresponsive NOD mice, β‐cells were depleted (Figures 1G,H and 3C,H). However, β‐cells were regenerated. Therefore, it was important to indicate the mechanism by which neogenic β‐cells appeared. Previously, we and others showed that neogenic β‐cells arise from neighboring α‐cells [41, 48, 49, 50]. Moreover, in a similar experimental setup in which chemically induced diabetic mice were treated with GABA, β‐cells were shown to increase in number from α‐cells [16]. Therefore, we generated Glucagon‐Cre driving eGFP mice, which were generated on a C57BL/6 background and backcrossed them for 10 generations into the NOD mouse strain, a 3‐year project (Figure 8C). These mice were maintained at 20 weeks of age, and nondiabetic mice were monitored for diabetes onset as described above.

eGFP+ α‐cells were detected in both nondiabetic and cured mice. A few β‐cells appeared in green in the cured mice (Figure 8B). However, most of the β‐cells were not green, indicating that β‐cell neogenesis was largely not from α‐cell progenitors.

As the majority of the neogenic β‐cells are not from an α‐cell origin, we stained for Ki67, a proliferation marker [51]. Ki67+ cells were found in the intestines of nondiabetic mice, but very few were found in the nondiabetic islet (Figure 8D,E, quantified in G and H). However, cured mouse islets are rich in Ki67+ cells, especially in the core of the islet, where the majority of β‐cells are found (Figure 8F, quantified in G and H), indicating that in the CT LADA model the leading mechanism of β‐cell neogenesis is replication.

## 3 Discussion

In this work, we present a strategy to cure LADA via the combination of GABA, SIT, and OMP. The combination of these drugs is considered a possible treatment for various diabetes types. Conflicting results were reported, and it was not clear if any combination of the drugs was effective. Our results here indicate that some requirements should be considered before CT administration is advised.

First, we found that CT is not applicable for rapid and acute development, as found in typical autoimmune diabetes, as observed in normal NOD mice, the leading T1D model. We were fortunate enough that, in our preliminary study, there was an old NOD mouse that exhibited a slow progression of the autoimmune disease. Thus, we developed and implemented a mouse model in which only nondiabetic NOD mice that remained normoglycemic beyond 20 weeks of age were included in this study. Mice that began developing diabetes after this age were defined as having LADA‐like disease. To further support our findings, we also examined the NZO mouse strain, which has been reported to develop a slowly progressing form of autoimmune diabetes and may therefore serve as an additional LADA model. However, in this strain, diabetes occurs only in a subset of males, while females remain normoglycemic [52, 53]. CT resulted in a bimodal response: ∼30% of the mice completely recovered (cured), whereas ∼70% did not respond to the treatment (unresponsive). For the 30% that responded, the effect was dramatic, leading to complete insulin independence and normoglycemia. We set the treatment window to 7 weeks, followed by a 7‐week follow‐up for cured mice. A statistically significant difference between cured and unresponsive mice was evident from Day 16 after treatment initiation (Figure 1B). For the remaining 33 days, unresponsive mice remained hyperglycemic. Notably, during this period, no mouse exhibited a delayed or gradual recovery in blood glucose levels. Thus, this finding demonstrates that the clear distinction between cured and unresponsive mouse populations reflects a genuine biological phenomenon.

The CT was accompanied by five control experiments. Untreated, GABA alone, GABA+SIT, GABA+OMP, and SIT+OMP (Figure S4). We included the two‐drug combinations as controls to determine whether one of the drugs could be omitted without compromising therapeutic efficacy. These control experiments showed that only the complete three‐drug combination produced a therapeutic effect, with a subset of animals responding to the treatment. As no diabetes recovery was observed in any of the two‐drug combinations, there was no rationale to proceed with single‐drug controls. The GABA experimental group is different. As it was previously reported that GABA alone could cure diabetes and lead to β‐cell regeneration [16], we still conducted this single drug administration to negate confounding effect if they exist.

Therefore, we aimed to identify what differentiates cured from unresponsive mice. Our aim was to identify an indicator that could be translated to identify CT‐responding LADA patients. Therefore, we focused on a simple blood test taken after LADA onset (but before treatment). A cfRNA test revealed that several markers are absent in responders. Two markers were found to be consistent in both the RNA‐seq and simple RT‐PCR test: Adgrb1 and Chd5.

We analyzed these genes using the UCSC GTEx V8 RNA‐Seq Read Coverage by Tissue tool [54]. According to this tool, the origin of the three genes is considered to be neuronal (Figure S9). However, the two genes Chd5 and Tmem266 were also found to be expressed in pancreatic β‐cells [55, 56]. In different studies, Adgrb1 was also found to be expressed in β‐cells [57]. Interestingly, the gene product of Adgrb1, BAI1, is a surface marker of apoptotic cells marking them for destruction by the immune system [58, 59]. The presence of RNA in cell‐free liquid biopsies indicates that a cellular population was damaged; thus, the RNA content reached the bloodstream [60]. Therefore, these findings are an indication of injured cell types that express these markers. It is most plausible that the presence of Adgrb1 and Chd5 cfRNA in the blood is an indication of a significant amount of destroyed β‐cells, or in other words, a fast‐progressing autoimmune attack on β‐cells. The fact that Adgarb1 was already defined as an apoptotic marker further highlights this possibility [58, 59]. Therefore, the absence of Adgrb1 and Chd5 implies that in CT‐cured mice, LADA is less advanced, not affecting cells that normally express Adgrb1 and Chd5 (most likely β‐cells) and did not yet passed the point of no return when autoimmune diabetes cannot be cured (at least, not with CT).

Our results can resolve a debate starting from 2017. Ben‐Othman et al. reported that chemically induced diabetes could be reversed in C57BL/6 mice [16]. However, von Herrath et al. reported that these results could not be replicated [61]. It could have been interesting to see what the levels of Adgrb1 and Chd5 were in mice of the two studies. It is possible that, in mice in the Ben‐Othman study, diabetes did not progress greatly and that Adgrb1 and Chd5 were not yet present in the blood.

Our model was not a chemically induced diabetes model and involved full autoimmune attack on the islets of Langerhans β‐cells (as shown in Figures 1, 2, and 3 as well as Figures 6, 7, and 8). Therefore, in this mouse study, GABA alone cannot alleviate diabetes and SIT and OMP are necessary (Figures 2 and S4).

When pancreata were analyzed, we were surprised to see that the islets were significantly larger in size (corresponding with the findings of Ben‐Othman et al. [16]) and were associated with insulitis. However, while insulitis was present, the islets were filled with β‐cells and had a normal morphology. There are two prevailing mechanisms described in the literature for β‐cell regeneration. β‐cell proliferation [62, 63, 64], and transdifferentiation from neighboring α‐cells [16, 48, 49]. To determine the origin of these β‐cells, we incorporated the Gcg–Cre driving eGFP, into the NOD background. The neogenic β‐cells were not from an α‐cell origin, as most β‐cells were eGFP negative. However, Ki67 staining indicated that substantial proliferation occurred in the cured mice, demonstrating that CT did not protect β‐cells against autoimmune attack (as was also shown by the fact that the mice were diabetic; Figure 1A) but that CT promoted β‐cell proliferation to regain normoglycemia. While these results highlight the importance of the β‐cell proliferation process in this model, it is still plausible that a preliminary event of another pancreatic progenitor cell transdifferentiation initiated the β‐cell neogenic process. In addition, the fact that the islets were larger in size in cured mice also supports the β‐cell proliferation hypothesis (Figure 3). β‐cell proliferation in certain diabetic stages was previously demonstrated to be possible [62].

An interesting point concerns what contributes to sufficient insulin secretion leading to normoglycemia in cured mice—whether it is related to increased β‐cell secretory capacity or the number of newly formed islets. In the in vivo experiment (Figure 2G; intraperitoneal GTT [IPGTT]), we measured the whole‐animal response, which does not distinguish between the number, size, or quantity of islets. Thus, this question could only be addressed in an in vitro experiment. Such an experiment would require extracting and hand‐picking islets according to size and quantifying the number of β‐cells per islet. While this complex approach is feasible in diabetes‐induced models, where mice can be pooled (such as in chemically induced models using STZ or alloxan), our experiments rely on the slow accumulation of individual mice that develop diabetes spontaneously. Therefore, this gradual pooling process would compromise the islets’ endocrine function over time. Hence, while this is an important question, we are currently unable to answer it.

The fact that in cured mice, islets were surrounded by insulitis was a remarkable finding. One would expect that if normoglycemia was regained, inflammation of the islet would have disappeared. However, insulitis persisted in the cured NOD mice even 7 weeks after normoglycemia was achieved. This phenomenon led us to question why β‐cells are not destroyed by insulitis. The fact that it is extremely challenging to isolate pancreatic leukocytes, and that leukocyte isolation via pancreas perfusion is impractical [65]; moreover, as we needed the pancreata for additional analyses, leukocyte characterization studies are limited to immunohistochemical studies. Cured mouse insulitis mainly consisted of T cells, and practically very few B cells or macrophages were found to populate the cured mouse insulitis. We reasoned that the T cells in the cured mice would be T‐regs; however, they were FOXP3 negative, indicating that the T‐cell population was not a classical T‐reg. Further investigation of the T‐reg lineage revealed that CT‐cured insulitis comprises largely Tr1 cells (Figure 7). We found that ∼80% of cured T cells coexpress IL‐10 and TGF‐β. Even if we consider that there is minimal overlap between the IL‐10+ and TGF‐β+ T‐cell populations in cured mice, we obtain a majority of 60–64% of the Tr1 cells within the T‐cell population. Such a large number of T‐regs potentially inhibits autoimmune attack on β‐cells. Therefore, a major finding of this study was to identify the Tr1 cell type as a desirable target and mechanism for suppressing the autoimmune attack.

Previously, in a chemically induced diabetes model, we showed that β‐cell ablation is not sufficient to induce β‐cell neogenesis and that the addition of inflammation (in our model, namely, pancreatic duct ligation and caerulein‐induced pancreatitis) is required [41, 48]. Later, we were able to map the biochemical pathway to the Par2 GPCR [50, 66]. Because persistent insulitis was found in cured mice, it may have been supported or was required to induce β‐cell neogenesis.

While our findings offer new insights into potential treatment avenues for LADA, several limitations should be acknowledged. First, this study was conducted exclusively in mouse models, and the degree to which these findings translate to human LADA remains uncertain. Second, although our identification of Adgrb1 and Chd5 as potential biomarkers is promising, the relatively small sample size in the cfRNA analyses limits the statistical power and generalizability of this result. Third, the mechanisms underlying the bimodal response to CT are still not fully understood, and further research is needed to uncover the factors determining responsiveness. Finally, although we demonstrated the presence of Tr1 cells in the insulitis of cured mice, functional validation of their immunosuppressive role in vitro was beyond the scope of this work.

In conclusion, our study provides valuable insights into the potential of CT for LADA treatment and highlights the importance of patient selection for successful outcomes in future clinical studies. The identification of Adgrb1 and Chd5 as potential blood markers for responders is a lead finding for developing a diagnostic tool to predict treatment efficacy. To translate these findings to human LADA patients, the first step will be to assess whether the bimodal distribution of Adgrb1 and Chd5 observed in the mouse model is replicated in humans. If the same pattern is seen, it would suggest that LADA patients with low Adgrb1 and Chd5 expression are likely to benefit from CT, making them strong candidates for this treatment. Alternatively, if the majority of LADA patients exhibit consistently low levels of these markers, it would indicate that CT could be broadly applicable to this patient population. On the other hand, if Adgrb1 and Chd5 are found to be highly expressed in human LADA patients, it would prompt further investigation. In this scenario, other biomarkers presented in Figure 5A and divided to biochemical pathways in Figure S10, could offer additional RNA markers for identifying patients who are more likely to respond to CT. Furthermore, our investigation into the origin of neogenic β‐cells sheds light on the mechanism of β‐cell neogenesis in the context of LADA. The presence of Tr1 cells in the insulitis of CT responders underscores their importance in achieving immune tolerance even amid ongoing autoimmune assault.

## 4 Materials and Methods

*Animals*: All mice were maintained in specific pathogen‐free conditions in the animal facility of the Azrieli Faculty of Medicine, Bar‐Ilan University, Safed, Israel. All animal experiments were conducted in accordance with institutional animal ethical committee guidelines (Permit Numbers: 91‐11‐2018 and 56‐08‐2021), which conform to the Guide for the Care and Use of Laboratory Animals published by the US National Institutes of Health (Eighth edition 2011). The animals were fed on a standard rodent chow diet and had access to tap water ad libitum. The mice were housed at a constant temperature and relative humidity under a regular light/dark schedule (12:12). Both male and female mice were used in the experiments.

Twenty‐week‐old NOD/LtJ (JAX #001976) males and females were monitored twice weekly for diabetes by measuring blood glucose levels via tail bleeding. Diabetes was defined as a blood glucose level ≥250 mg/dL on 2 consecutive days. A total of 95 mice were randomized into the following treatment groups: 26 mice received CT, eight mice received GABA, seven mice received a combination of GABA+SIT, eight mice received a combination of GABA+OMP, seven mice received a combination of SIT+OMP, and seven mice were untreated diabetics. Additionally, 13 mice were used for circulating cfRNA analysis, and four mice were used in a Cre–GFP experiment. A separate cohort of 15 NZO mice was included, with six untreated NZO mice and nine NZO mice receiving CT. Mice received (0.3 mg/g/day) GABA (A2129‐25G; Sigma‒Aldrich, St. Louis, MO, USA) in phosphate‐buffered saline (PBS; 02‐023‐1A; Biological Industries, Beit‐Haemek, Israel) or (0.03 mg/g/day) SIT (PHR1857‐1G; Sigma‒Aldrich) or (0.02 mg/g/day) OMP (PHR1059‐1G; Sigma‒Aldrich) in dimethyl sulfoxide (D8418‐100 mL; Sigma‒Aldrich) or vehicle by oral gavage daily for 7 weeks. The different dosages regiments were previously determined and established safe for rodents [67, 68, 69].

Lantus (insulin glargine; Sanofi‐Aventis Deutschland, Germany) was injected subcutaneously into diabetic mice with a blood glucose ≥400 mg/dL to maintain normoglycemia and to minimize death rates in the experimental and control groups. Insulin doses were adjusted depending on the measured blood glucose levels.

*Intraperitoneal GTT*: IPGTTs were performed at the end of the experiment. After a 16‐h fast, 2 g/kg body weight of a solution of 20% D (+)glucose anhydrous (V6M758206N; CARLO ERBA, Germany) was injected intraperitoneally into the mice. Blood was drawn from the tail vein, and blood glucose levels were measured at 0, 30, 60, 90, 120, 150, and 180 min after the injection.

*Immunofluorescence staining*: Tissue was fixed in 4% formaldehyde (4% in PBS) overnight, incubated overnight in 30% sucrose (S0389‐5KG; Sigma–Aldrich), embedded in optimal cutting temperature (20248; Bar Naor Ltd., Israel) and frozen at −80°C. Slides 5 µm thick were washed three times with PBS and treated with 0.3% Triton (194845; MP Biomedicals, USA) in PBS for 15 min. After washing with PBS for 15 min, the slides were incubated in blocking solution supplemented with 5% normal donkey serum (Jackson ImmunoResearch, USA) for 40 min at room temperature. The slides were incubated overnight at 4°C with primary antibodies against insulin (1:100; polyclonal guinea pig anti‐insulin CA95051; Dako, USA), glucagon (1:400; rabbit anti‐glucagon PAb; NBP2‐38330; Novus Biologicals, USA), and somatostatin (1:400; rat mAb anti‐somatostatin (YC7); Novus Biologicals). The secondary antibodies used were labeled with Alexa Fluor 488 (711‐545‐152), Alexa Fluor 647 (706‐605‐148), and rhodamine red (712‐295‐153) (all from Jackson ImmunoResearch). Nuclei were visualized with DAPI Fluoromount‐GTM (17984‐24; Bar Naor Ltd.).

*Ki67 staining*: The slides were washed three times with PBS and treated with 0.3% Triton in PBS for 15 min. After washing with PBS for 15 min, the slides were boiled in 10 mM citrate (pH = 6) for 15 min, kept at room temperature for 15 min and then washed in PBS for 2 × 15 min. The staining procedure was continued as described above.

*Immune cell staining*: The slides were incubated overnight at 4°C with conjugated secondary antibodies against CD45 (1:100, rat, D124203; Novus Biologicals), CD19 (1:50, mouse, D124224; Novus Biologicals), CD3 (1:200, rat, FAB4841G; R&D, USA), F4/80 (1:200, rat, D124213; Novus Biologicals), and FOXP3 (1:100, mouse, 42‐5773‐82; Thermo Fisher Scientific, USA). Nuclei were visualized with DAPI Fluoromount‐GTM. IL‐10 and TGFβ were stained with primary antibodies (1:150, mouse; sc‐365858; Santa Cruz Biotechnology, USA; and 1:100, mouse; sc‐130348; Santa Cruz Biotechnology) and incubated with secondary antibodies as described above.

*Image acquisition*: The slides were imaged using AxioScan. Z1 microscope (Zeiss, Jena, Germany). Quantification of immunofluorescence images was performed using ImageJ. For FOXP3, images were acquired by a Leica SPi 8 Super‐Resolution gSTED Inverted Confocal Microscope (Wetzlar, Germany).

*Processing of whole blood*: Blood samples were collected in EDTA‐anticoagulated vacutainers from the tails of the mice at the start of the experiment. Blood samples were centrifuged at 1500×*g* for 20 min at 4°C. The plasma was then stored at −80°C until RNA isolation.

*cfRNA isolation*: Total RNA was isolated from 50 µL of serum using the Circulating Nucleic Acid Kit (55114; QIAGEN, Germany) according to the manufacturer's protocol. To digest trace amounts of contaminating DNA, RNA was treated with 10× Baseline‐ZERO DNase. DNase I‐treated RNA samples were purified and further concentrated using RNAclean and concentrator‐5 (74204; QIAGEN) according to the manufacturer's instructions. The final eluted RNA was stored immediately at −80°C.

*Library preparation*: We prepared stranded RNA‐Seq libraries using a Clontech SMART‐seq Stranded Kit (634862; Takara Bio, USA) according to the manufacturer's instructions. For cDNA synthesis, we used option 2 (without fragmentation), starting from highly degraded RNA. The input of 7 µL of RNA sample was used to generate cDNA libraries suitable for next‐generation sequencing. For the addition of adapters and indexes, we used the Takara dual index kit‐Indexing Primer Set HT for Illumina V2‐12 (634416; Takara Bio). The unique indexes of each 5′ PCR primer were subsequently added to each sample to distinguish pooled libraries from each other. The amplified RNA‐seq library was purified by immobilization onto an AMPure XP PCR purification system (Beckman Coulter). The library fragments originating from rRNA and mitochondrial rRNA were treated with ZapR v2 and R‐Probes according to the manufacturer's protocols. For final RNA‐seq library amplification, 14 cycles of PCR were performed, and the final 20 µL was eluted in Tris buffer following amplified RNA‐seq library purification. The amplified RNA‐seq library was stored at −20°C prior to sequencing.

*Sequencing, data processing, and quality control*: For each sample, more than 20 million paired‐end reads were sequenced using an Illumina NextSeq or HiSeq sequencer.

4.1

*Quantitative RT‐PCR*: RNA was isolated from the plasma of cured and unresponsive mice and were transcribed into cDNA using High‐Capacity RNA‐to‐cDNA Kit (4387406; ThermoFisher Scientific, USA) and analyzed by RT‐PCR using Fast SYBR Green Master Mix (4385612; ThermoFisher Scientific). Oligonucleotides were designed using the Primer3 PCR Primer Design Tool according to the RNA‐Seq data. Primers are listed in Table S1. Differential expression was calculated using the equation of 2^(−ΔΔCt)^, with GAPDH as an endogenous control.

*Statistical analysis*: Statistical analysis was performed with GraphPad Prism 5.0. The data are presented as the mean ± SEM, and the differences between groups were analyzed by ANOVA and *T* tests. The results were considered to be significantly different at *p < 0.05, **p < 0.01, and ***p < 0.005.

## Author Contributions

W.S., S.E., S.P., and R.P. designed the research studies. W.S., S.E., A.D., N.A., M.K., and M.A. conducted the experiments. W.S., A.D., S.E., N.A., A.S., M.K., M.A., A.M., S.P., and R.P. acquired the data and analyzed it. R.P. provided the reagents. W.S., A.D., and R.P. wrote the manuscript. S.E. and A.D. produced similar amounts of data and therefore are listed as contributed equally. S.P., the head of the genomic center, is the corresponding address for the genomic analysis and data, while R.P. is the head of the regenerative medicine and diabetes laboratory and is the corresponding address for all the experimental procedures. W.S., A.D., S.E., N.A., A.S., M.K., M.A., A.M., S.P., and R.P. have read and approved the final manuscript.

## Funding

This study was funded by the Russell Berrie Galilee Diabetes SPHERE.

## Conflicts of Interest

The authors Wisal Sawaed, Sivan Eliyahu, Shira Perez, and Ron Piran are named inventors on a patent related to this work (US 63/973,302; filed on February 1, 2026). All other authors declare no conflicts of interest.

## Ethics Statement

All mice were housed under specific pathogen‐free conditions at the Azriely Faculty of Medicine's Animal Facility, Bar‐Ilan University, Safed, Israel. All the experiments were conducted according to the institutional animal ethical committee guidelines, which conform to the Guide for the Care and Use of Laboratory Animals published by the US National Institutes of Health (Eighth edition 2011). Permit Approval Numbers: 91‐11‐2018 and 56‐08‐2021

## Supporting information



Supporting file 1: mco270813‐sup‐0001‐figures.pdf

## Data Availability

All data generated during and/or analyzed during the current study are available from the corresponding author on reasonable request.
