# Supplementary material for: Markers Predicting Cure With Combinatorial Treatment in a Mouse Model of Latent Autoimmune Diabetes in Adults
Source: MedComm (2020). 2026 Jul 12;7(7):e70813. doi: 10.1002/mco2.70813 (PMC13357692; doi:10.1002/mco2.70813)
Supplement: Supplementary file 1 — Supporting file 1: mco270813‐sup‐0001‐figures.pdf [file MCO2-7-e70813-s001.pdf]

# Supplementary information to: "Markers Predicting Cure with Combinatorial Treatment in a Latent Autoimmune Diabetes in Adults Mouse Model".

Wisal Sawaed[1], Ahmad Dallasheh[1#], Sivan Eliyahu[1#], Nura Aburomi[1], Aviad Sivan[2], Marina Kurtz[2], Michael Assa[3], Assaf Malka[1], Shira Perez[2*], and Ron Piran[1*].

**Figure S1**

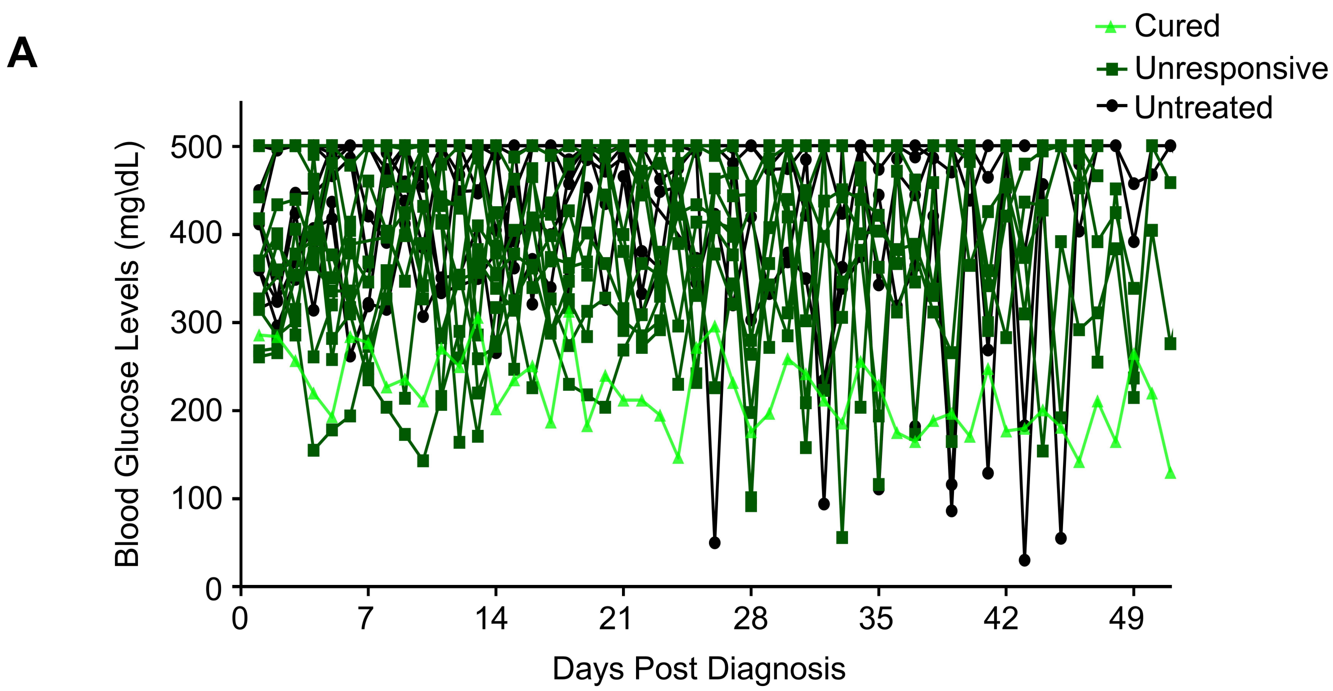
A
Cured
Unresponsive
Untreated
Blood Glucose Levels (mg\dL)
500
400
300
200
100
0
0
7
14
21
28
35
42
49
Days Post Diagnosis

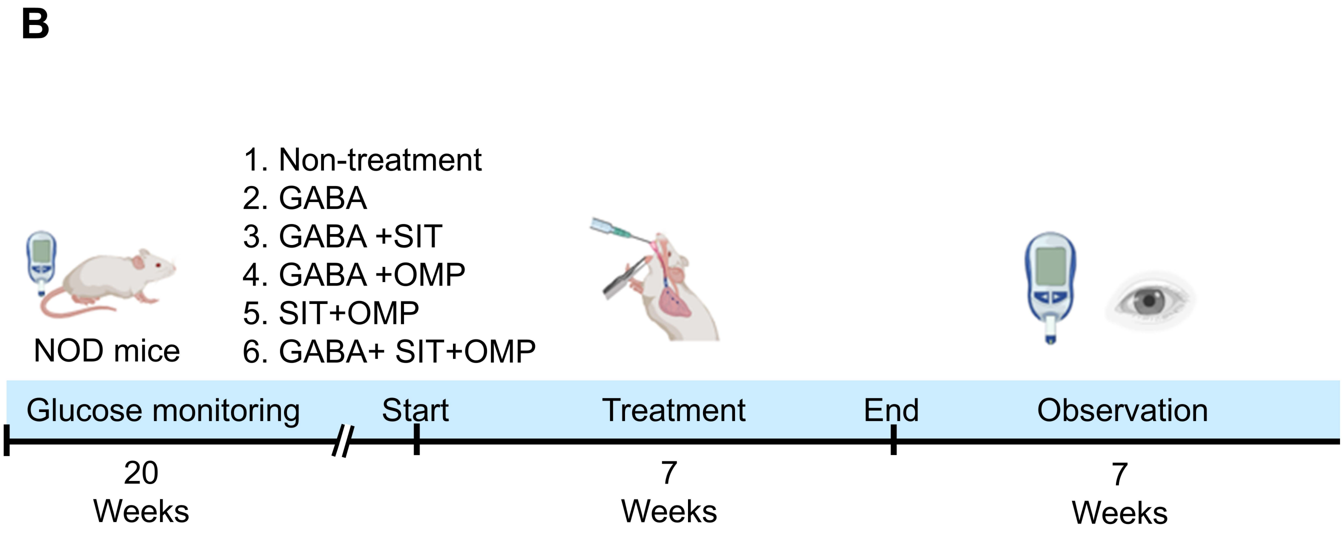
B
1. Non-treatment
2. GABA
3. GABA +SIT
4. GABA +OMP
5. SIT+OMP
6. GABA+ SIT+OMP
NOD mice
Glucose monitoring
Start
Treatment
End
Observation
20
Weeks
7
Weeks
7
Weeks

**Figure S1. Old NOD mice may recover from autoimmune diabetes following CT.**

**A**. Glycemic levels of individual mice administered with or without CT. Black lines indicate untreated mice, Green lines indicate mice treated with CT. Dark green lines represent mice which did not respond to the treatment. A light green line indicates a single NOD mouse (30 weeks of age) treated with CT, showing long-term, full, and complete recovery from diabetes. **B.** Schematic illustration of the experimental design.

Figure S2

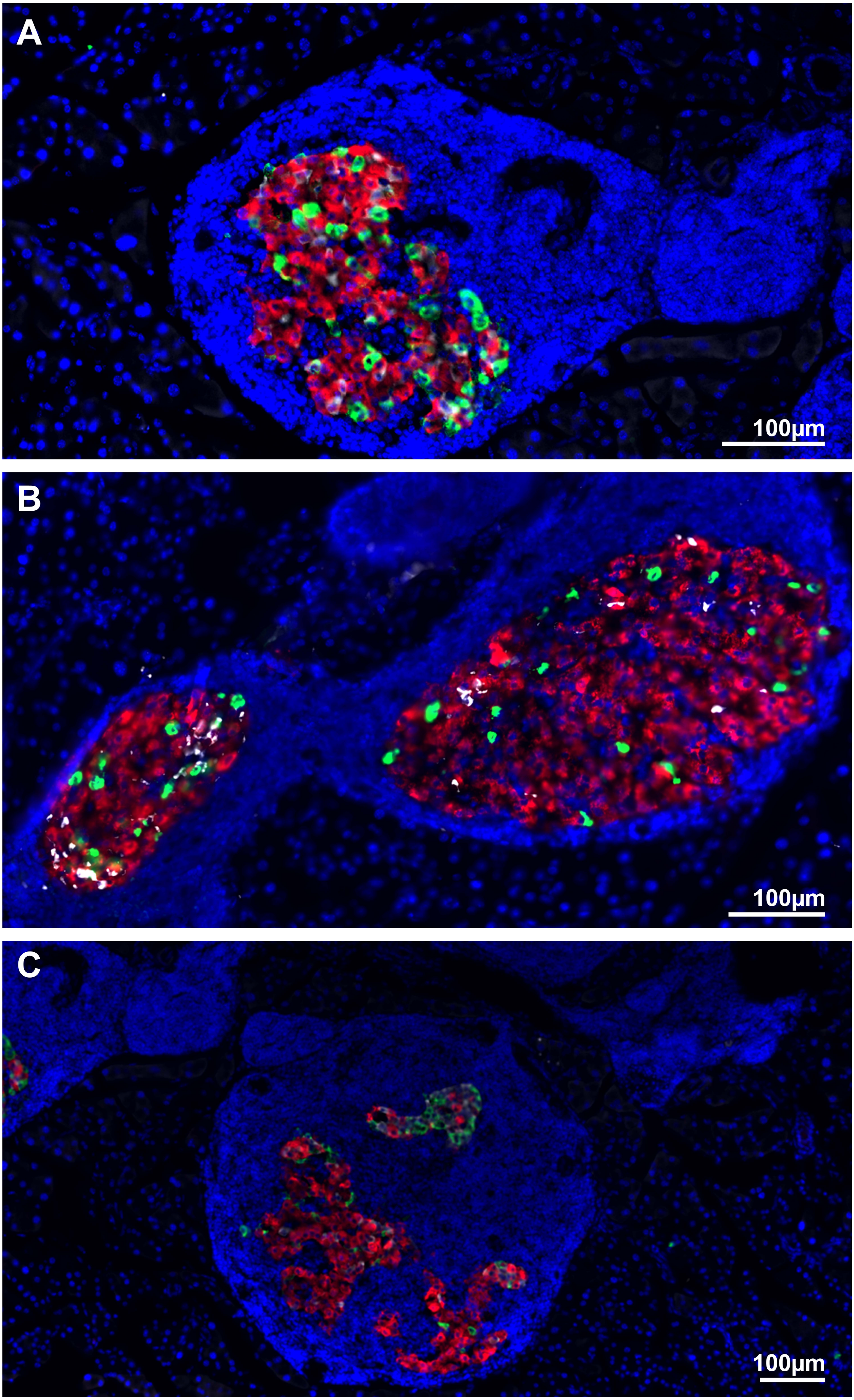

**Figure S2. Full-size islets of CT-cured mice from the main article. A.** A full-size image of a cured mouse presented in Figure 3B. **B, C.** Full-size images of the $INS^+$ islets from cured mice presented in Figure 3G. Insulin in red, glucagon in green, and somatostatin in white. Scale bars are 100μm.

**Figure S3**

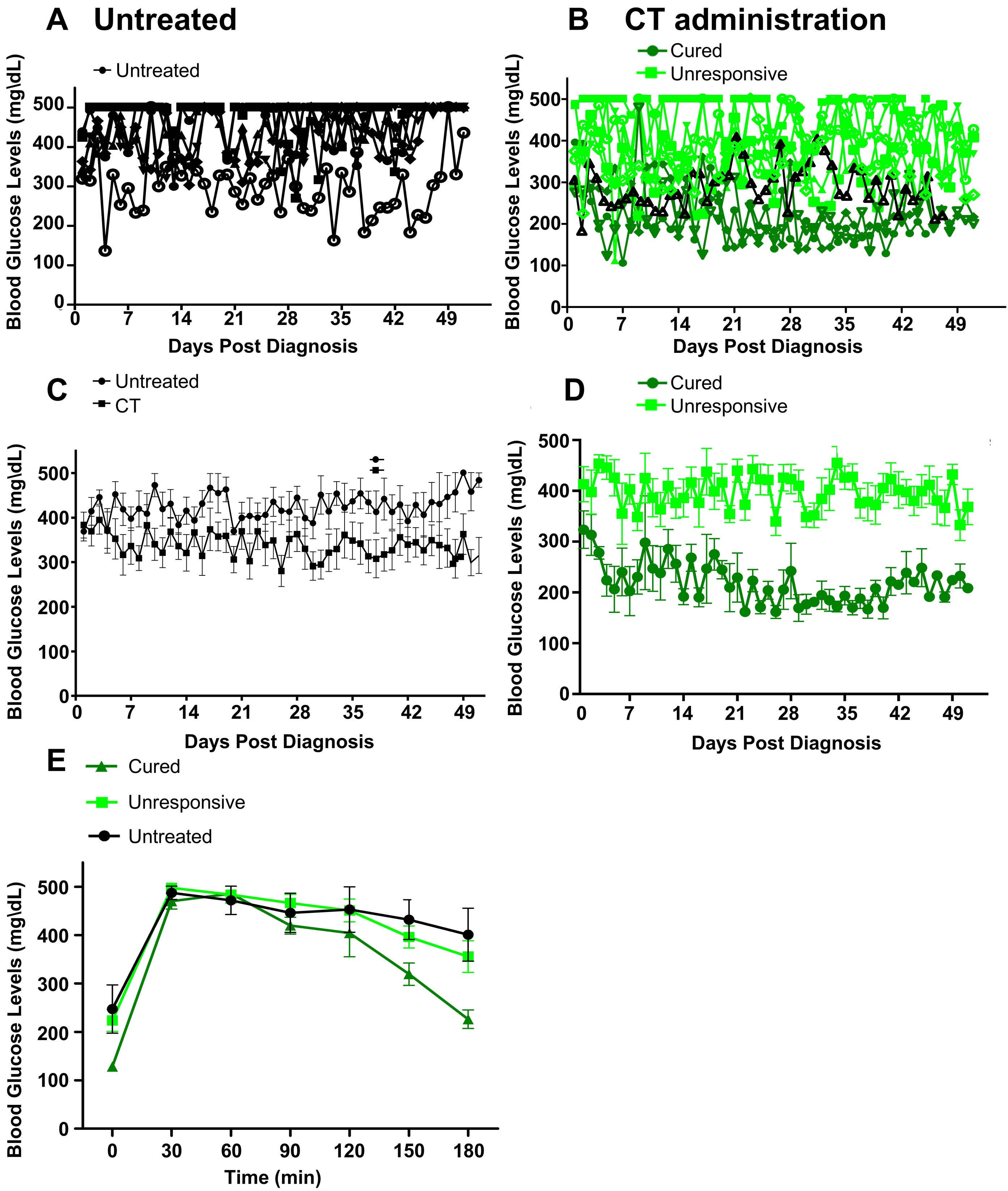
A Untreated
Untreated
Blood Glucose Levels (mg\dL)
Days Post Diagnosis
B CT administration
Cured
Unresponsive
Blood Glucose Levels (mg\dL)
Days Post Diagnosis
C
Untreated
CT
Blood Glucose Levels (mg\dL)
Days Post Diagnosis
D
Cured
Unresponsive
Blood Glucose Levels (mg\dL)
Days Post Diagnosis
E
Cured
Unresponsive
Untreated
Blood Glucose Levels (mg\dL)
Time (min)

**Figure S3. NZO mice recover from autoimmune diabetes similarly to NOD mice.**

**A.** Real-time glycemic levels of individual untreated mice (n=6). **B.** NZO mice administered with CT (n=9), where dark green indicates cured (n=3), light green indicates Unresponsive mice to CT (n=6), and the black line is the glycemic level averages of all the mice that received CT. **C.** The glycemic level averages of CT administered **(B)** Vs. control **(A)** groups. **D.** The glycemic level averages of the two groups (cured and unresponsive mice) presented in **B. E.** IPGTT performed in mice reaching the end of a 7 week-long treatment. Data are mean ± standard error of the mean (SEM).

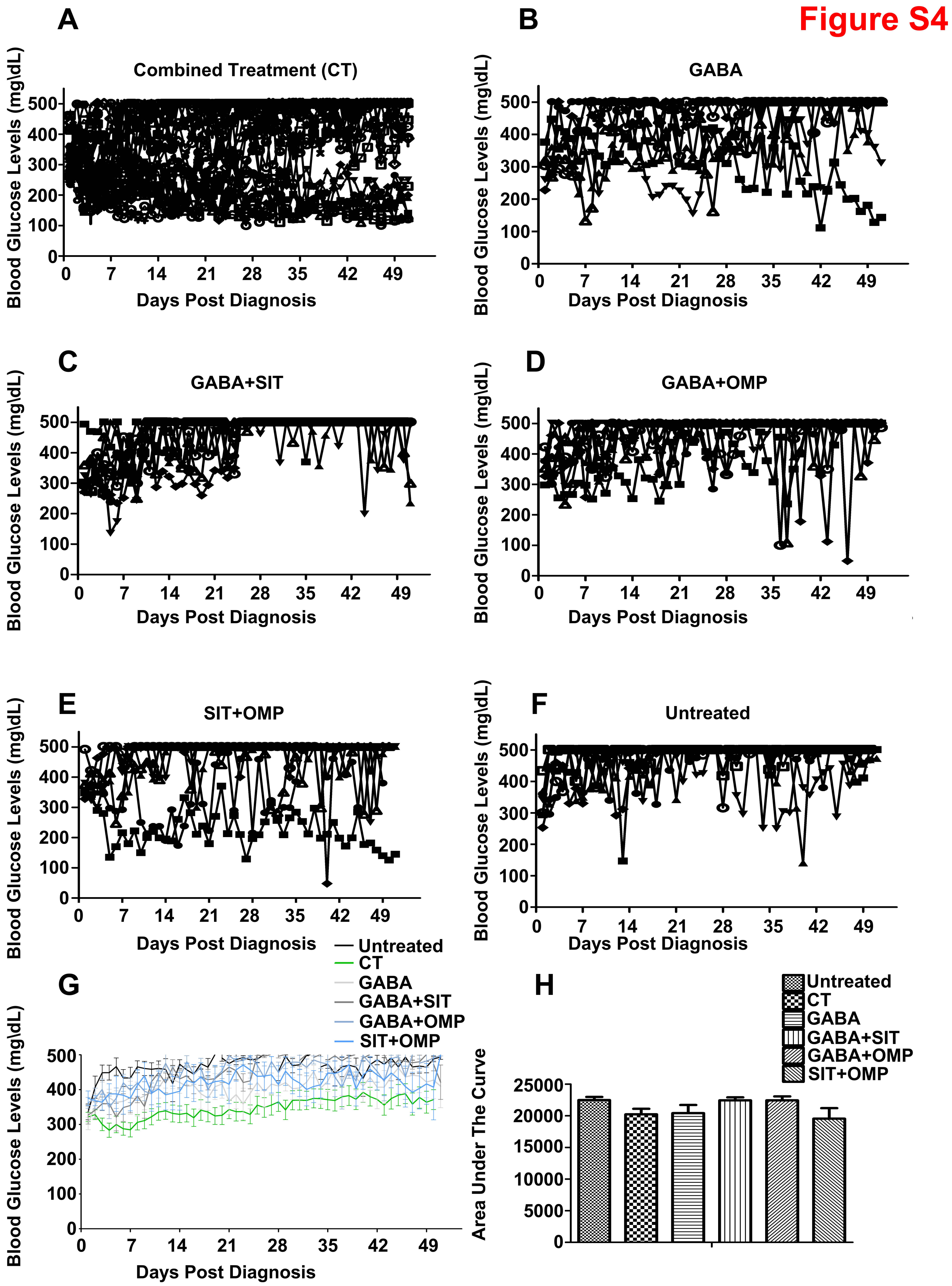
Figure S4
A
Combined Treatment (CT)
B
GABA
C
GABA+SIT
D
GABA+OMP
E
SIT+OMP
F
Untreated
G
H
Blood Glucose Levels (mg\dL)
Days Post Diagnosis
Area Under The Curve
Untreated
CT
GABA
GABA+SIT
GABA+OMP
SIT+OMP

**Figure S4. Only the combination of GABA, SIT, and OMP may lead to normoglycemia. A.** The same data appearing in Fig.1A. **B.** 8 animals treated with GABA alone. **C.** 7 animals treated with GABA+SIT. **D.** 8 animals treated with GABA+OMP. **E.** 7 animals treated with SIT+OMP. **F.** 7 untreated animals. Each line represents one mouse. **G.** average glycemic levels of the different treatment groups **(A-F)**. **H.** Average area under the curve of the different treatments. Note that for **G** and **H** the CT group includes both the cured and unresponsive mice. Error bars are SEM.

# Figure S5

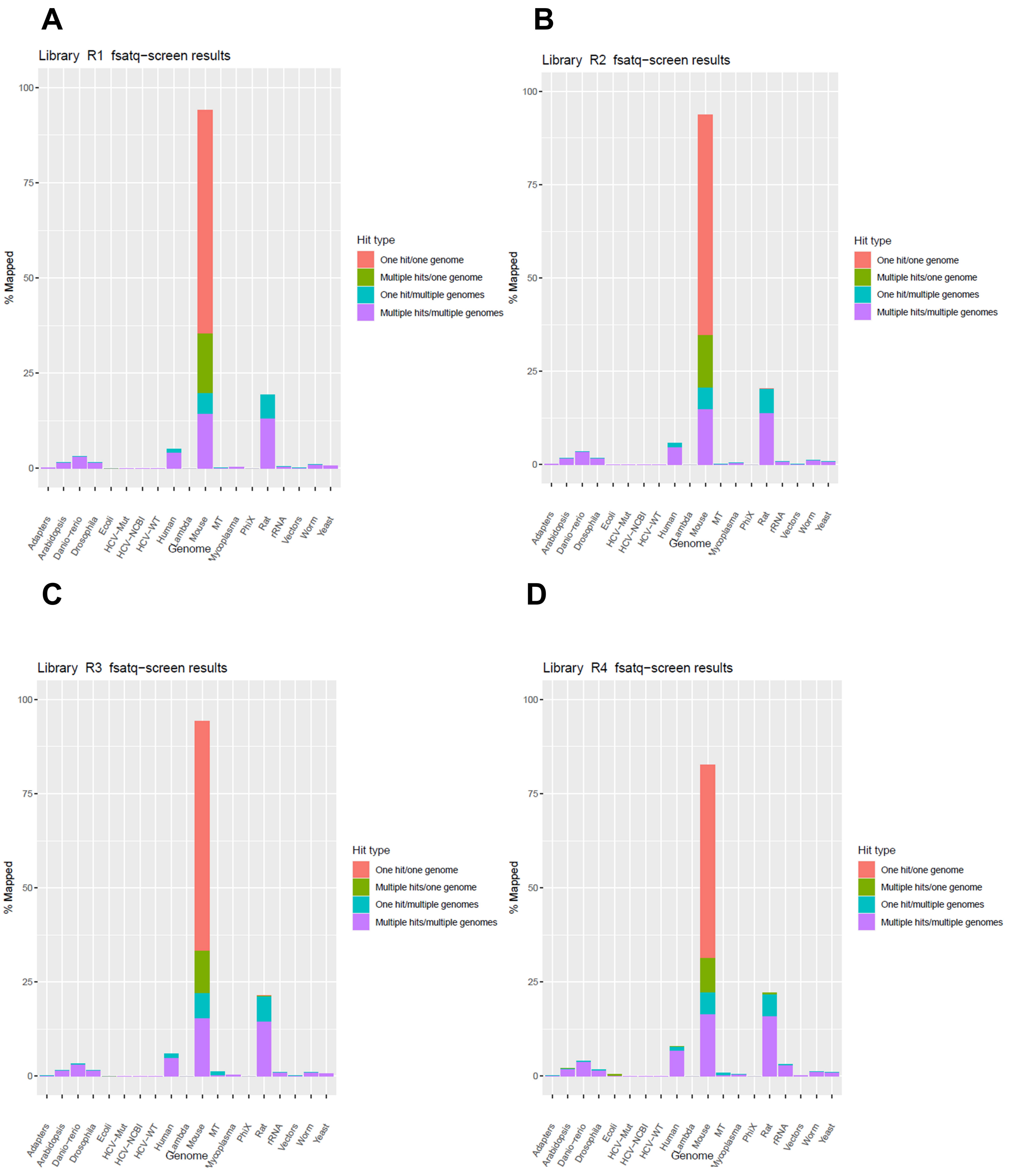
A
Library R1 fsatq−screen results
B
Library R2 fsatq−screen results
C
Library R3 fsatq−screen results
D
Library R4 fsatq−screen results
% Mapped
Genome
Adapters
Arabidopsis
Danio−rerio
Drosophila
Ecoli
HCV−Mut
HCV−NCBI
HCV−WT
Human
Lambda
Mouse
MT
Mycoplasma
PhiX
Rat
rRNA
Vectors
Worm
Yeast
Hit type
One hit/one genome
Multiple hits/one genome
One hit/multiple genomes
Multiple hits/multiple genomes

**Figure S5. Fastq-screen analysis confirms that the isolated RNA from mice R1-R4 (A-D, respectively) are of a mouse origin.** Stacked bar plots showing the percentage of mapped reads for each genome, with most uniquely mapped reads (red) aligning to the mouse genome.

**Figure S6**

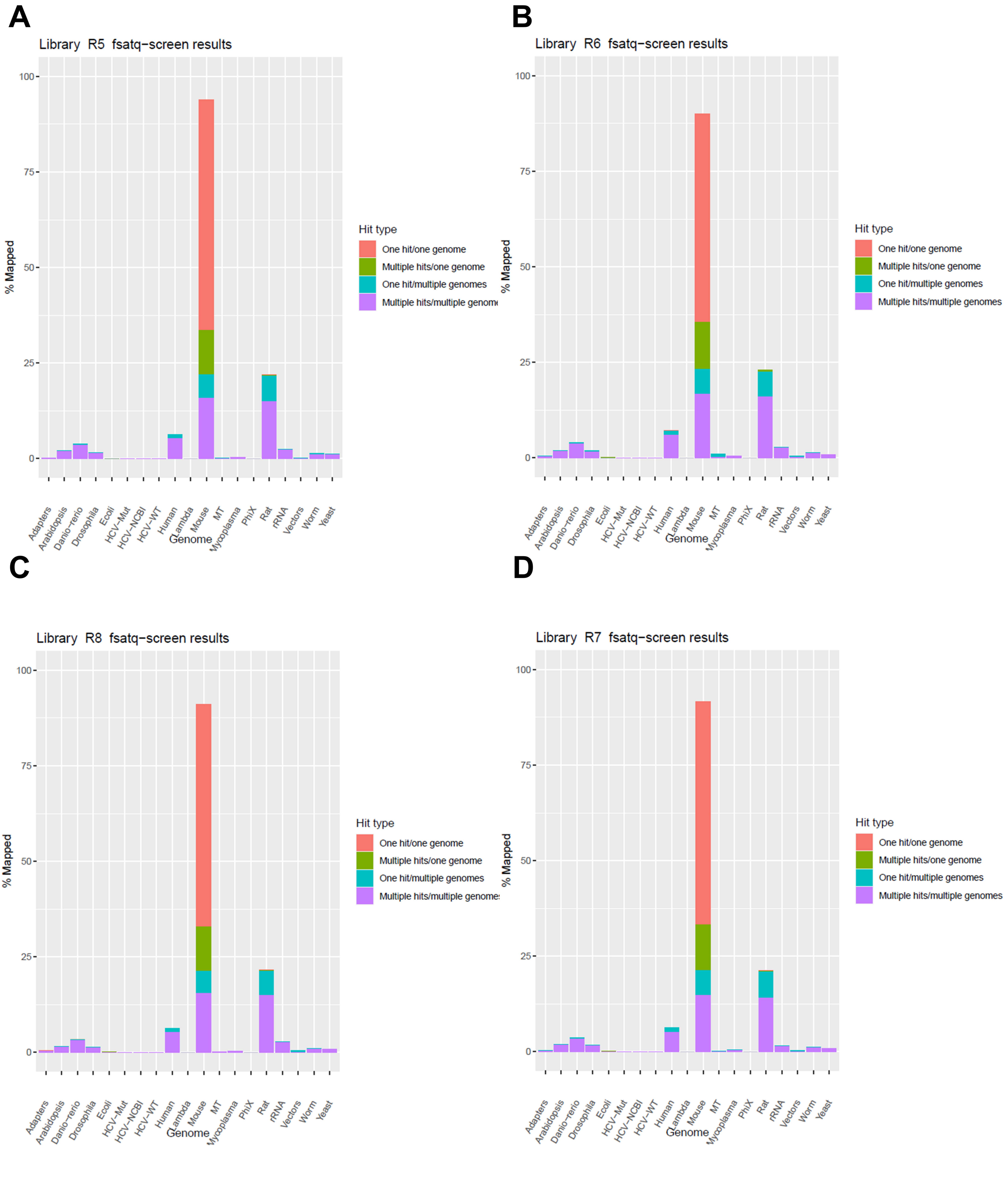
A
Library R5 fsatq−screen results
B
Library R6 fsatq−screen results
C
Library R8 fsatq−screen results
D
Library R7 fsatq−screen results
% Mapped
0
25
50
75
100
Genome
Adapters
Arabidopsis
Danio−rerio
Drosophila
Ecoli
HCV−Mut
HCV−NCBI
HCV−WT
Human
Lambda
Mouse
MT
Mycoplasma
PhiX
Rat
rRNA
Vectors
Worm
Yeast
Hit type
One hit/one genome
Multiple hits/one genome
One hit/multiple genomes
Multiple hits/multiple genomes

**Figure S6. Fastq-screen analysis confirms that the isolated RNA from mice R5-R8 (A-D, respectively) are of a mouse origin.** Stacked bar plots showing the percentage of mapped reads for each genome, with most uniquely mapped reads (red) aligning to the mouse genome.

# Figure S7

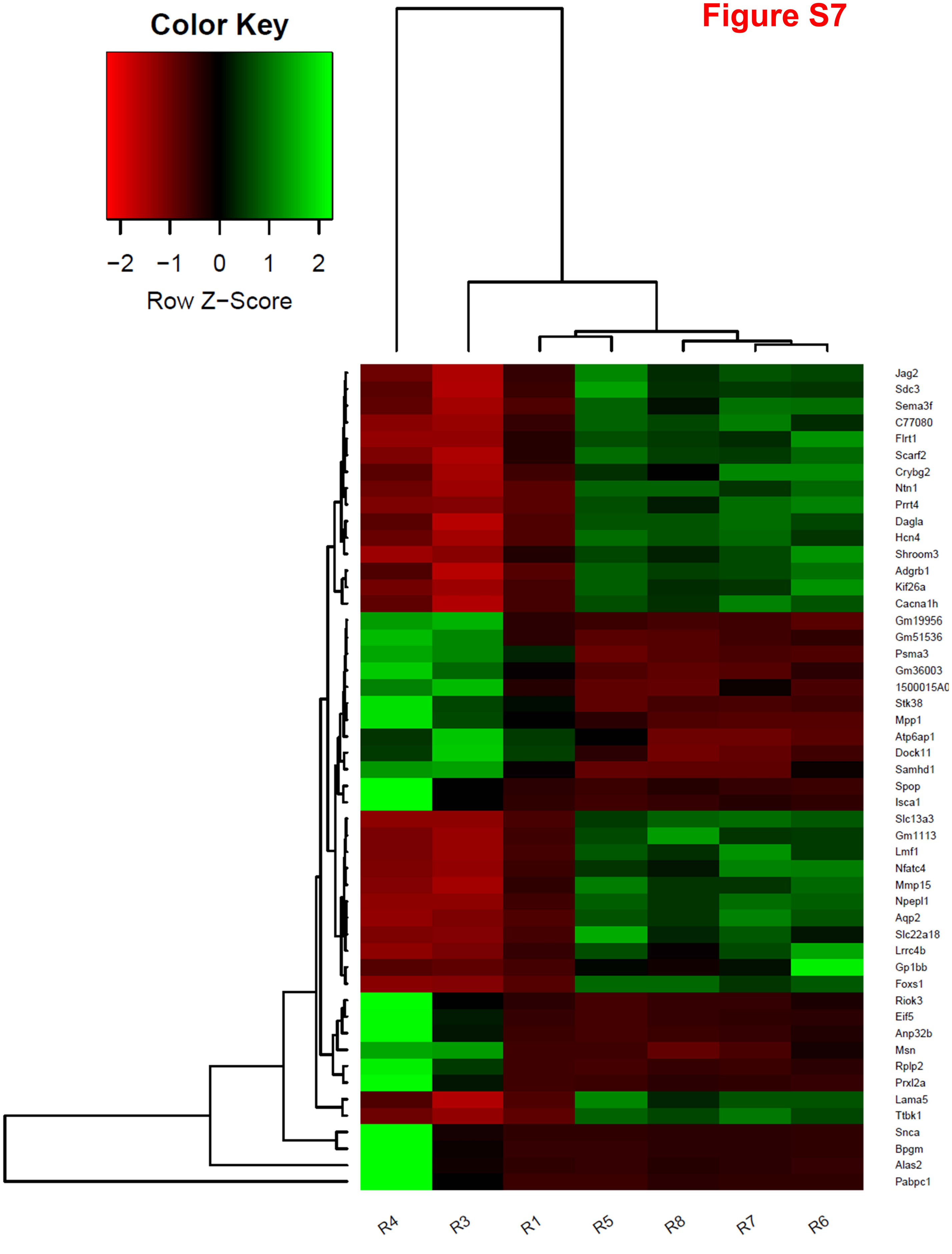
Color Key
−2 −1 0 1 2
Row Z−Score
Jag2
Sdc3
Sema3f
C77080
Flrt1
Scarf2
Crybg2
Ntn1
Prrt4
Dagla
Hcn4
Shroom3
Adgrb1
Kif26a
Cacna1h
Gm19956
Gm51536
Psma3
Gm36003
Stk38
Mpp1
Atp6ap1
Dock11
Samhd1
Spop
Isca1
Slc13a3
Gm1113
Lmf1
Nfatc4
Mmp15
Npepl1
Aqp2
Slc22a18
Lrrc4b
Gp1bb
Foxs1
Riok3
Eif5
Anp32b
Msn
Rplp2
Prxl2a
Lama5
Ttbk1
Snca
Bpgm
Alas2
Pabpc1
R4
R3
R1
R5
R8
R7
R6

**Figure S7. A detailed, full-size image of the heatmap analysis presented in Figure 4D.** This heatmap visualizes the top 50 DE genes between cured and unresponsive mice, showing the expression patterns of the genes across samples, clustering together genes that have correlated expression patterns. The heatmap clusters samples based on the Euclidean distance between the expression values.

# Figure. S8

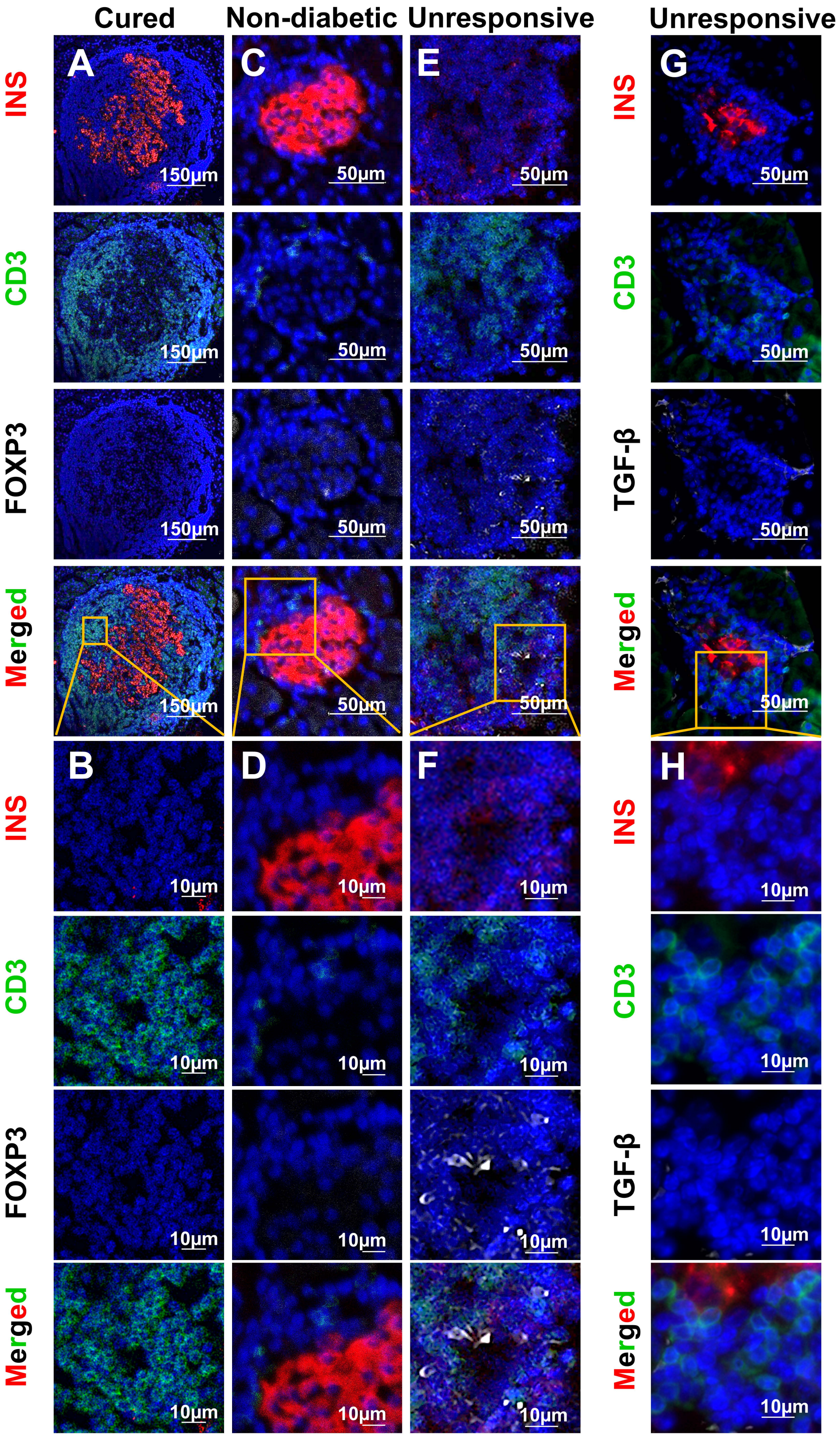
Cured
Non-diabetic
Unresponsive
Unresponsive
A
C
E
G
B
D
F
H
INS
CD3
FOXP3
TGF-β
Merged
150μm
50μm
10μm

**Figure S8. Complementary images for Figure 7.** Representative immunofluorescence images of pancreatic islets, showing insulin staining in red and CD3, a T-cell marker staining in green, FOXP3 staining in white(**A-F**), and TGF-β staining in white (**G-H**). **A** and **B.** Low-magnification **(A)** and high-magnification **(B)** of the full-size islet shown in Figure 7A and B, from a cured mouse. No $FOXP3^{+}$ cells were found in the cured islets. **C.** and **D.** A low magnification **(C)** and high magnification **(D)** of an islet from a non-diabetic mouse. **E.** and **F.** A low magnification **(E)** and high magnification **(F)** of an islet from an unresponsive mouse. **G** and **H.** A low magnification **(G)** and high magnification **(H)** of an islet from an unresponsive mouse. Note that most of the $CD3^{+}$ T-cells are also TGF-$\beta^{-}$ cells. Scale bars in A=150µm, B, D, F, H=10µm, and C, E, and G=50µm.

Figure. S9

A

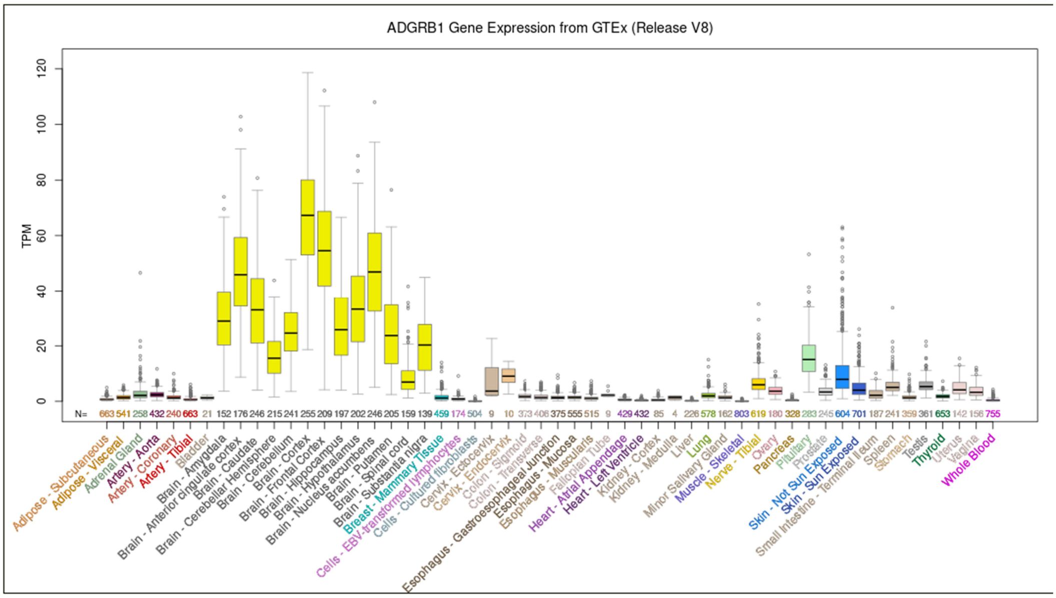


B

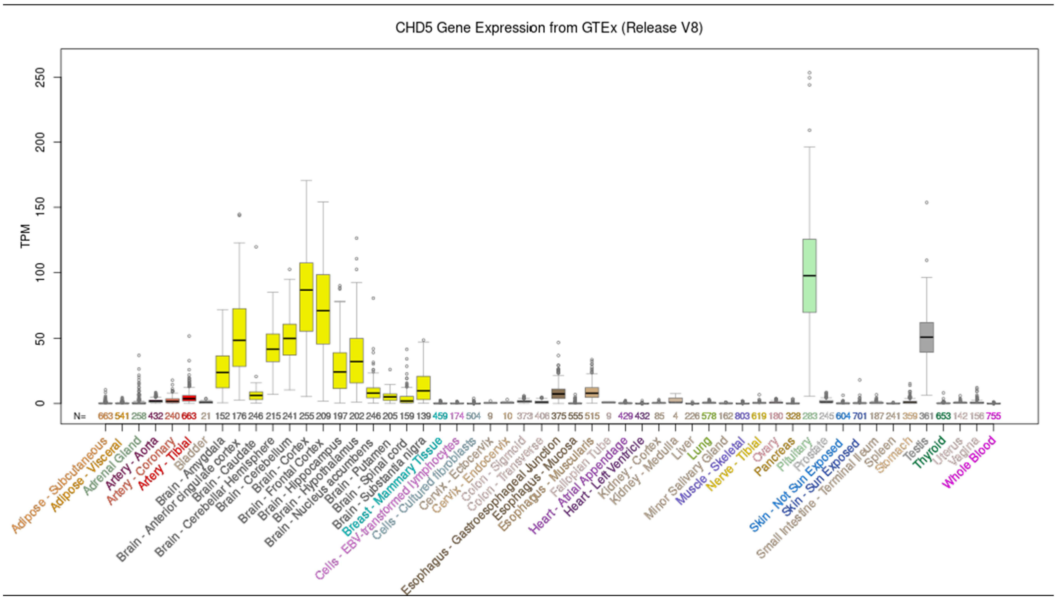


C

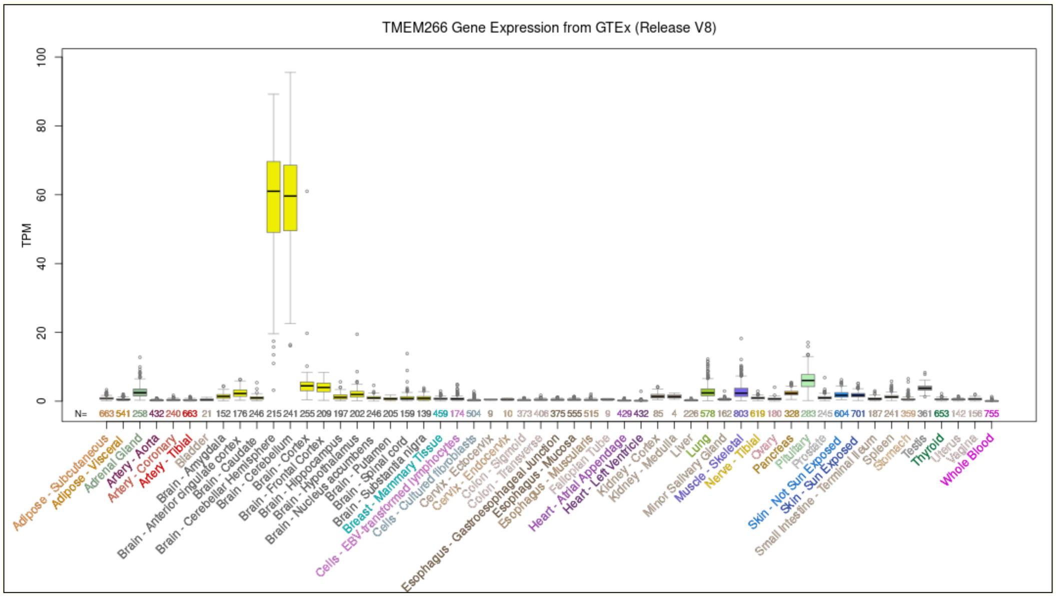

**Figure S9. The selected Adgrb1, Chd5, and Tmem266 genes are mainly expressed in neuronal tissues**. The selected genes were analysed using the UCSC GTEx V8 RNA-Seq Read Coverage by Tissue tool. **A.** For the Adgrb1 gene. **B.** For the Chd5 gene, and **C.** For the Tmem266 gene. note that according to this tool, the origin of the three genes is from a neuronal origin.

# Figure S10

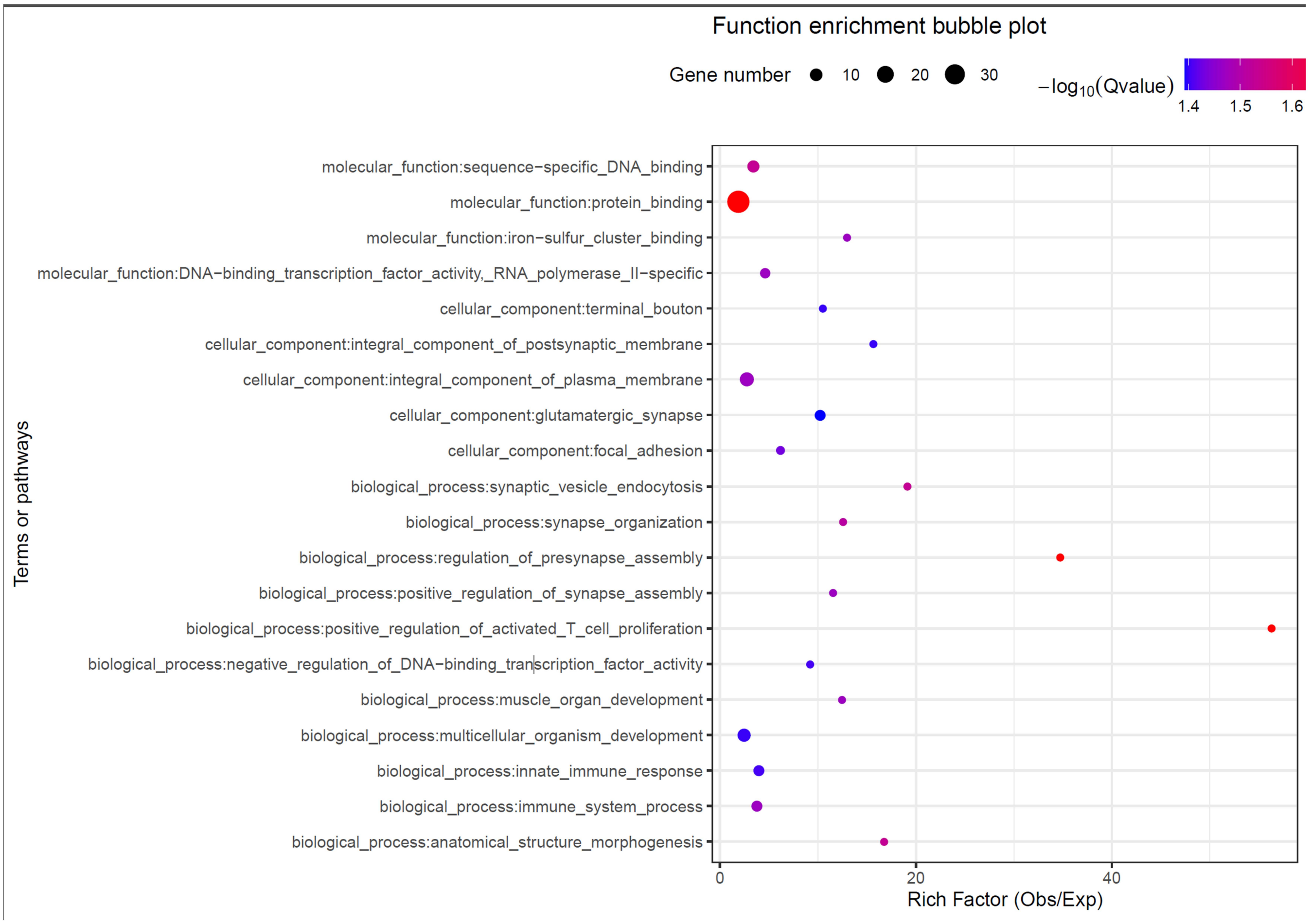


**Figure S10. Function enrichment bubble plot based on GO term ID, with Rich Factor.** Observed Gene Num/Expected Gene Num (Observed Gene Num: Observed gene number of this term/pathway in the customized gene set, Expected Gene Num: Expected gene number of this term/pathway in the customized gene set). Q value = [ -1 * log10(adjP) ].

# Primer list

| Gene | Primer sequence (5'->3') |
| --- | --- |
| Adgrb1 For | CGCTCTTGGGAACGTCTCG |
| Adgrb1 Rev | GACTTGATCCAGCGACTTGC |
| Chd5 For | TCAGGCCCCTTATTGCCAAG |
| Chd5 Rev | CCTTAAACGGGTTGTTGGCG |
| Tmem266 For Pair 1 | CCAAAGAGAAGGGTCTGACTGG |
| Tmem266 Rev Pair 1 | ACTGTTGAGACTTGCACTGAGC |
| Tmem266 For Pair 2 | ACCAAAGAGAAGGGTCTGACTG |
| Tmem266 Rev Pair 2 | CACTGAGCAAAAATACCTGCC |
| Tmem266 For Pair 3 | ACCAAAGAGAAGGGTCTGACT |
| Tmem266 Rev Pair 3 | CAGGAAACTGTTGAGACTTGC |
| Gapdh For | AACTTTGGCATTGTGGAAGG |
| Gapdh Rev | ACACATTGGGGGTAGGAACA |

**Table S1. The primers used to verify Adgrb1, Chd5, and Tmem266.** Note that for Adgrb1 and Chd5 statistically significant results were obtained (Figure 5C). For Tmem266 variation was large. An attempt to replace the primers to pairs 2 and 3 did not improve our results. The Gapdh primers are established markers adopted from the literature:

[1] M. Mehdi, R. Krawetz, Y. Zhang, J. B. Rattner, A. Godollei, H. J. Duff, D. E. Rancourt "Impact of stirred suspension bioreactor culture on the differentiation of murine embryonic stem cells into cardiomyocytes," BMC cell biol. 12, (2011).

[2] C. Toda, T. Shiuchi, H. Kageyama, S. Okamoto, E. A. Coutinho, T. Sato, Y. Okamatsu-Ogura, S. Yokota, K. Takagi, L. Tang, K. Saito, S. Shioda, Y. Minokoshi, "Extracellular Signal–Regulated Kinase in the Ventromedial Hypothalamus Mediates Leptin-Induced Glucose Uptake in Red-Type Skeletal Muscle," Diabetes 62, no. 7 (2013): 2295-2307.

[3] C-X. Wang, T. Ma, M-Y. Wang, H-Z. Guo, X-Y. Ge, Y. Lin, "Facile distribution of an alkaline microenvironment improves human bone marrow mesenchymal stem cell osteogenesis on a titanium surface through the ITG/FAK/ALP pathway," Int J Implant Dent 7, no. 56 (2021).
